# Digital restoration and feature recognition of a Qing-Dynasty vernacular dwelling based on multimodal data fusion

**DOI:** 10.1038/s41598-025-31544-7

**Published:** 2025-12-13

**Authors:** Xiaotang Xia, Zhihan Chen, Yulin Xu

**Affiliations:** School of Urban Construction Engineering, Wuhan University of Science and Technology, Wuhan, 430065 China

**Keywords:** Vernacular architecture, HBIM (historic building information modeling), Multimodal data fusion, SLAM laser scanning, Cultural feature recognition, Knowledge visualization, Digital heritage preservation, History, History, Mathematics and computing

## Abstract

This study addresses the urgent challenge of digitally preserving severely damaged vernacular architecture that lacks complete historical documentation. Taking the Dan Tao’s Former Residence, a Qing Dynasty dwelling in the Jingchu region, as a case study, we propose a reproducible multimodal framework for reverse restoration. The approach integrates SLAM-based laser scanning, UAV photogrammetry, historical documentary evidence, analogy-driven HBIM construction, and knowledge-graph visualization. By bridging the semantic gaps between material remains, images, and textual records, the workflow enables high-fidelity digital modeling of complex components while decoding cultural features at multiple scales. Results demonstrate that the framework overcomes limitations of insufficient point-cloud density and missing documentation, achieving accurate 3D restoration of degraded structures and establishing a scalable cultural feature recognition system for Jingchu vernacular architecture. This research provides both methodological innovation and practical tools for conservation, offering a transferable paradigm for safeguarding endangered architectural heritage worldwide.

**Supplementary Information:**

The online version contains supplementary material available at 10.1038/s41598-025-31544-7.

## Introduction

Architectural heritage, as a fundamental component of cultural heritage, embodies rich historical, cultural, and artistic connotations, serving as an indispensable treasure of human civilization^1^. It not only encapsulates the unique cultural essence of local regions but also serves as a vital channel for preserving beliefs, documenting local characteristics, and recording social norms and customs, thereby playing a pivotal role in the continuous progression of human society^2,3^. However, due to the impact of natural changes and the evolution of social cultures, many architectural heritage sites have suffered significant damage and are now under threat of extinction^4^. With digital technology integrating ever more profoundly into every facet of economic and social development, cultural transmission and innovation are encountering new opportunities^5^. In this context, China has been actively enhancing policy support for the digital preservation of architectural heritage. As of March 1, 2025, the “Cultural Relics Protection Law of the People’s Republic of China” will officially incorporate digital preservation within its mandate, clearly stipulating the use of digital technologies for recording, archiving, researching, and presenting cultural relics. This legislative measure offers the first direct legal foundation for the digital preservation of architectural heritage and other cultural relics at the national level. In light of this context emerges the Jingchu region, centered around the ancient city of Jingzhou, renowned for a 5000-year history of urban development and stands as the cradle of Chu culture. The vernacular architecture techniques in Jingchu are uniquely ingenious, employing methods such as guiding order, hiding and revealing, winding paths, and emphasizing concealment to convey emotions and meaning, thus forming a distinctive architectural language^6^. Nevertheless, due to rapid urbanization process and the disruption of historical textures, many traditional buildings and ancient towns within the Jingchu region have been subjected to reckless remodeling despite repeated bans. There is thus a pressing need to employ modern information technologies to digitally preserve, restore, and rejuvenate these sites.

With the advancement of technology, the approaches for data collection and research in architectural heritage digital preservation projects have become increasingly diverse^7^, especially in the detailed collection of architectural information. Guo et al. focused on the Sansu Shrine Garden, obtaining high-precision point cloud data by integrating ground laser scanning and digital photogrammetry^8^. Lin et al. used the Faro S350 laser scanner, combined with SCENE software, to acquire and process point cloud data of the architectural complex in Fenghuang Village^9^. This highlights the growing significance of integrating digital tools such as laser scanning, drone photography, and 3D point clouds, along with other digital resources, in the architectural information collection for cultural heritage^10,11^. These technologies allow for the comprehensive preservation of information from severely damaged historical sites^12^. This study applies Simultaneous Localization and Mapping (SLAM) laser scanning technology to survey Qing Dynasty vernacular dwellings in the Jingchu region. SLAM 3D laser scanning technology, equipped with a built-in inertial navigation system, enables high-precision synchronization and stitching. Its portability and flexibility make it particularly suitable for data collection of complex structures, facilitating the simultaneous capture of architectural details such as murals and decorations. The efficiency and quality of modeling achieved are notably superior compared to traditional technologies^13^.

In recent years, there has been rapid advancement in the research of methods and applications for architectural heritage restoration and reconstruction, with a diversification of research topics. For example, Lin et al. implemented heritage building information modeling (HBIM) to create 3D point cloud models for the Church of San Stefano in Volterra, Italy, and utilized virtual reality (VR) to conduct restoration, resulting in a detailed and comprehensive 3D model of the scene^14^. Similarly, Chen et al. examined ancient watchtower architectural groups in China’s Tibetan regions as a case study, merging two types of data captured by drones and 3D laser scanners to generate 3D models. By analyzing the models and combining similar residual conditions, they proposed a virtual restoration strategy, which culminated in the construction of a 3D restoration model^15^. Lin et al. applied point cloud slicing technology to automatically extract the contour of the ancient Giant Wild Goose Pagoda in China, proceeding with detailed modeling based on these extracted outline features from the reconstructed point cloud. This was followed by block stitching and texture mapping, resulting in a complete and lifelike pagoda model^16^. Liu et al. conducted error analysis and accuracy assessment based on factors such as scan station alignment accuracy, point cloud model reliability, point cloud data noise, and point cloud data layering. This enabled the precise acquisition of 3D point cloud model data for Dacheng Palace and the creation of a realistic spatial model of the heritage building^17^. However, current research exhibits significant limitations. Most existing studies are focused on 3D digital modeling of well-preserved architectural heritage, while systematic technological approaches for reverse reconstruction combining multi-source data for endangered ancient buildings with structural damage exceeding level III are yet to be established. Some restoration studies face restrictions due to insufficient point cloud density for decorative components, limiting the precision of detail restoration. Additionally, in the absence of comprehensive historical documentation, these studies often rely solely on stylistic inferences from contemporaneous architecture, leading to restoration accuracy challenges.

Knowledge visualization, an emerging research field that has evolved from data and information visualization, leverages visual representations to facilitate the dissemination and innovation of collective knowledge. It encompasses a variety of graphical methods capable of constructing and conveying complex knowledge^18^. Research on architectural heritage knowledge visualization transforms material remains, construction techniques, and cultural connotations into interactive, multidimensional information carriers through digital technologies^19^, providing an innovative path for cultural heritage protection and transmission. This approach has now been applied to various fields, including cultural heritage studies and the protection of ancient architecture. Zhang et al. employed knowledge visualization theory by integrating digital protection resources to delve into the architectural and opera culture of ancient stage platforms. They used visual methods, such as animation and AR/VR, to digitally revitalize Shanxi’s ancient stage platforms, thus preserving elements of traditional Chinese culture^20^. Shou integrated “Constructivist” thinking into spatial subjects by developing three-dimensional typological diagrams to analyze the spatial structure of Huizhou vernacular architecture. This approach highlighted the interconnectedness and organizational order of various elements, systematically unveiling the flexible potential of these typical “Enclosure” forms in spatial construction logic^21^. Another study by Zhang et al. involved creating a layered knowledge database and developing a VR interactive system, allowing users to “Touch” architectural details^22^. Xue et al. examined 17 existing ancient stage platforms in the Huizhou region, providing an in-depth analysis of architectural features across dimensions like floor plan layout, spatial composition, structure, decoration, and wooden frameworks. They systematized and visualized these features into an “Architectural Gene Map” to offer fresh insights and models references for preservation efforts^23^. However, current research on knowledge visualization in architectural heritage protection is hampered by an unclear cultural identification path, limiting the depth of cultural connotation exploration.

The Dan Tao’s Former Residence in Anfeng Village, Chibi City, exemplifies challenges in protecting Qing Dynasty Jingchu vernacular dwellings. This city-level traditional building deeply reflects the ceremonial order of clan society through its spatial organization, and features like braced-tie wooden frameworks and carved eaves preserve the essence of traditional construction techniques. However, long-term natural erosion and structural aging have significantly compromised its load-bearing system. Issues like wall cracks exceeding 60%, a collapsed roof, and loose mortise-and-tenon joints severely weaken the building’s overall stability, with current safety indicators falling far below the requirements for cultural heritage protection.

In summary, this study contributes to the field in three key ways: (1) it develops a systematic multimodal framework for reverse restoration of highly damaged vernacular dwellings using SLAM-based 3D acquisition, analogy-driven HBIM construction, and cultural feature recognition; (2) it bridges the semantic gap between material evidence, images, and textual records through knowledge-graph integration, enabling multidimensional cultural decoding; and (3) it establishes a scalable visual knowledge atlas of Jingchu vernacular architecture, offering new pathways for digital preservation and heritage management.These contributions collectively advance existing HBIM-based heritage reconstruction methodologies by introducing a more integrated multimodal workflow, a reproducible semantic-linking mechanism, and a scalable cultural–knowledge representation strategy.

The remainder of this paper is organized to demonstrate the application and effectiveness of multimodal HBIM and cultural gene visualization in the digital preservation of Qing Dynasty vernacular architecture. Sect. “Materials and methods” introduces the study area and details the multimodal data acquisition methods. Sect. “Overview of the study area” presents the results of data processing, knowledge acquisition, and HBIM reconstruction. Sect. “Technical methods” provides an in-depth discussion of architectural characteristics and cultural ideology, highlighting how semantic interpretation and cultural feature mapping advance beyond conventional HBIM approaches. Finally, Sect. “Multimodal data fusion” summarizes the methodological contributions and practical implications of this study, offering a replicable paradigm for heritage conservation, restoration planning, and sustainable use of traditional architecture.

## Materials and methods

### Overview of the study area

**Fig. 1:**
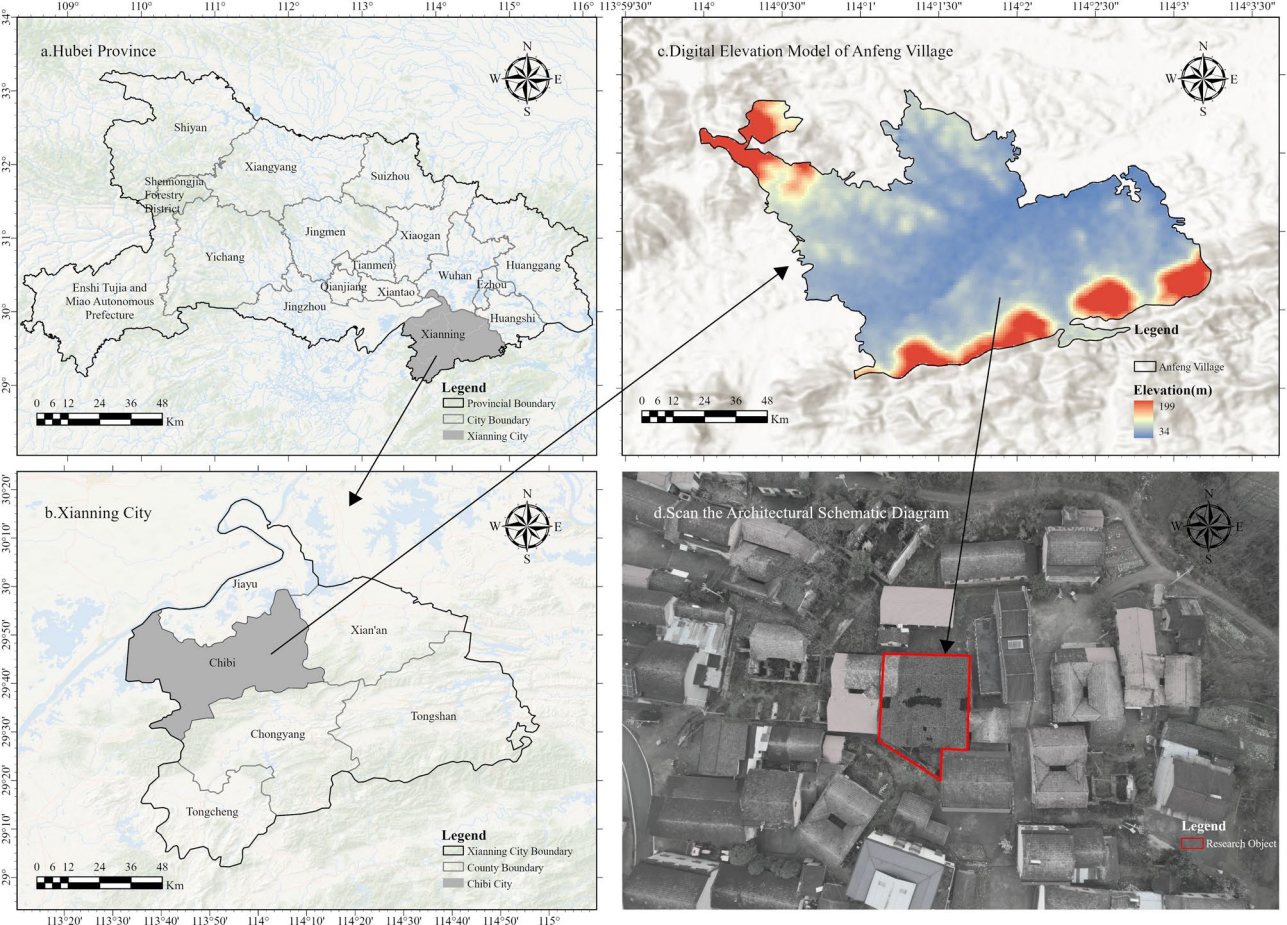
Research area information.

Anfeng Village, located in the northeastern part of Chibi City, Hubei Province, is recognized as a nationally significant traditional village (sixth batch)^24^. The Dan family architectural complex, situated in Anfeng Village, Zhonghuobu Town, serves as a prominent example of Qing Dynasty Jingchu vernacular dwelling, preserving the typical historical features and cultural essence. The complex dates back over 200 years ago in the Qing Dynasty, covering a site of 39,600 square meters, with a constructed area of approximately 1391 square meters. The complex consists of eight independent courtyards, ten skywells, and 63 rooms. The buildings are set with bluish-gray stone slab foundations, fired blue bricks, and lime mortar using “Four-Seven Construction Techniques”. The roofs were covered with blue-black tiles, creating the Hui-character-shaped courtyard. The skywells and corridors connect various functional areas, balancing both practicality and symbolic significance. The exterior windows mainly feature “Single and Double Coin” motifs in stone-carved lattice, while the inner windows and courtyard-walls feature wood carvings of auspicious designs such as “Sun and Moon Shining Together” and ”Deer and Cranes in Harmony”. These elements reflect the homeowner’s social status and adhere to hierarchical norms. Additionally, the carvings on the stone doorposts follow the hierarchical architectural norms of the family. The architectural design of the Dan family complex is deeply influenced by the space philosophy of “Returning to Vitality” and their core ethical values of “Loyalty, Filial Piety, Brotherhood, and Trust”. The well-preserved Qing Dynasty furniture, daily utensils, stone wells, and Feng Shui layouts—particularly those related to Fengxiang Spring Creek—serve as cultural snapshots of the agrarian society in the Jingchu region. These elements represent both the material and spiritual culture of the time, shaping the rural identity of the region. The detailed location of the study area is shown in Fig. 1.

### Technical methods

**Fig. 2:**
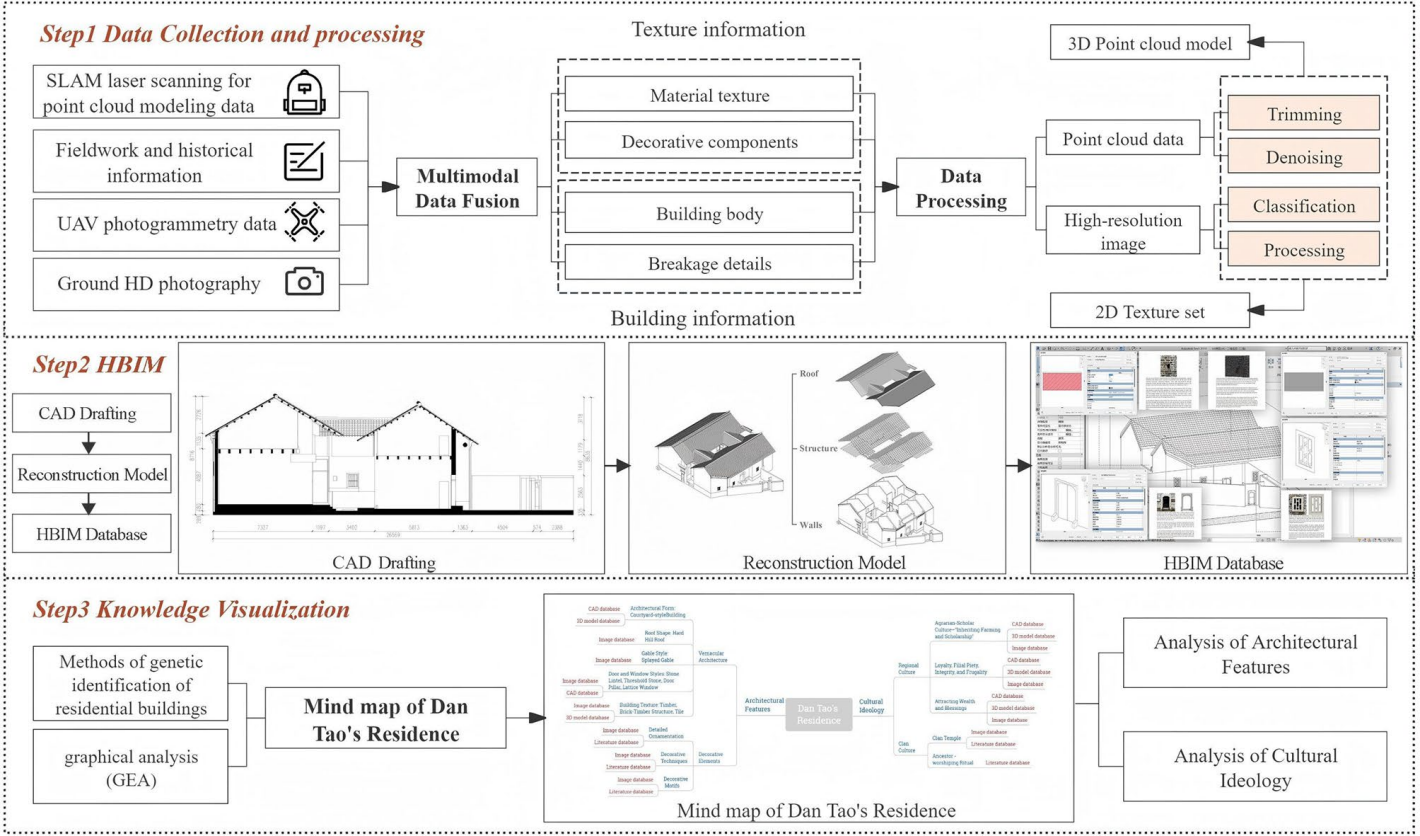
Research area information.

The proposed method mainly involves three steps (Fig. 2). (1) Multimodal Data Collection and Fusion. High-precision 3D point cloud data were collected through SLAM laser scanning, UAV photogrammetry, and ground-based HD photography. Structural and textural attributes—including material properties, decorative elements, and damage patterns—were fused into a semantically labeled multimodal database. (2) Model Reconstruction. Architectural frameworks were reconstructed using CAD-based technical drafting and SketchUp (SketchUp Pro 2022) parametric modeling, explicitly addressing structural degradation and missing features. The workflow integrated historical reference alignment to infer original geometries in severely damaged parts, generating a structured CAD database and a 3D model repository with annotated degradation states. (3) Knowledge Visualization. Cultural semantics and spatial-decorative relationships were analyzed through vernacular gene identification and graphical analytical methods. The visual knowledge atlas and 3D repository systematically decoded the building’s historical significance, emphasizing adaptive conservation strategies for degraded heritage.

### Multimodal data fusion

1. Field investigation and historical data.

This study employed a longitudinal field investigation approach to research the Dan Tao’s Former Residence located in Anfeng Village, Chibi City, conducted from February 2025 to May 2025. The research aimed to trace the construction date, historical background, and cultural lineage of the Dan family, while also documenting architectural deterioration and evidence of adaptive modifications. Historical data, including local chronicles, government archives, family history documents, and historical images, were collected and analyzed. Additionally, folk materials, including genealogical records and inscriptions, were collected during field visits. Through comparative analysis, the historical records in local chronicles were compared with contemporary documentation material to identify changes and continuities. This comparative analysis confirmed the building’s age, the craft features outlined in the literature, and the alterations observed in the Dan Tao’s Former Residence during the collectivization era.

(2) 3D laser scanning to obtain point cloud model data.

GoSLAM T100Pro RTK laser scanner was used for on-site scanning to acquire point cloud and imagery data from historical structures, with the data recorded in both the WGS84 and CGCS2000 coordinate reference systems. The GoSLAM Mapping Master desktop software and GoSLAM LidarWorks processing software were employed to assist in the operation and post-processing of the data, enabling the generation of point cloud data enriched with RGB color information. Following the scanning process, In the multimodal data fusion stage, the registration of datasets was performed using the Iterative Closest Point (ICP) algorithm. The GoSLAM system integrates LiDAR and IMU, enabling a SLAM-based workflow that maintains high-accuracy spatial positioning and performs real-time trajectory correction during mobile scanning. This configuration provides stable cross-scene alignment without requiring GNSS assistance. Therefore, ground control points (GCPs) or check points were not necessary for ensuring coordinate consistency across datasets, as the SLAM-ICP workflow maintained coherent spatial alignment throughout the scanning and processing phases.Real-Time Kinematic (RTK) technology was utilized to precisely ascertain the absolute coordinates of control points with high accuracy.

(3) UAV aerial photography data.

Unmanned Aerial Vehicle (UAV) photogrammetry technology was employed to acquire comprehensive spatial data about the Dan Tao’s Former Residence and its surrounding environment. In this study, the DJI AIR 2S UAV was employed to capture aerial imagery of the building’s roof, courtyard, and surrounding area from a vertical perspective and four oblique angles, allowing for a holistic view of the structure and its surroundings^25^.

(4) High-resolution image capture

Professional-grade photographic equipment was used to capture high-resolution images of the building, with images at a resolution of 24.2MP, to extract detailed texture data of the building’s main structure and decorations. These images were subsequently used for texture mapping optimization. Additionally, satellite remote sensing was employed to capture 3D geographical data of the residence’s surrounding environment, including terrain, geographical features, and vegetation coverage.

In summary, point cloud data provided spatial coordinates and structural dimensions, UAV imagery covered the macro layout of the building, and high-resolution images focused on micro-level details. These data sources collectively constructed a comprehensive building element database. By integrating multimodal data, this study primarily obtained architectural information in three specific areas: decorative components, material textures, and damage details.

### HBIM construction

HBIM is an extension of Building Information Modeling (BIM). Compared with traditional BIM, HBIM incorporates not only geometric and parametric information but also historical documentation, material typologies, construction chronology, deterioration conditions, and other conservation-related metadata specific to heritage buildings. In this study, the HBIM model integrates semantic attributes such as material categories, historical phases, conservation status, and relationships among structural and decorative components. The attribution of the datasets to HBIM components was implemented through customized Revit shared parameters linked to external thematic databases. Field survey information, historical documents, 3D laser-scanning point clouds, UAV images, and archival records were first organized into databases describing material typologies and decay conditions, historical phases and construction evolution, and cultural or symbolic meanings. These datasets were imported into Revit and bound to specific components through shared parameters and parameter-mapping scripts, allowing each geometric instance to retrieve corresponding semantic records from the external tables. This parameter-based structure not only enriches the HBIM with cultural and conservation metadata but also enables query-based filtering, deterioration assessment, and phase-specific analytical operations, extending its functionality beyond visualization. In addition, the implementation of cultural semantic–component association is based on a structured linking mechanism in which each Revit component contains a shared parameter that stores a unique identifier. This identifier is used to retrieve corresponding cultural semantic records—such as symbolic interpretations, archival descriptions, or historical narratives—from external thematic databases. When a URL-type parameter is used, the identifier dynamically directs the component to a specific document path in the database, ensuring that cultural information remains externally maintained, consistently referenced, and automatically synchronized across components. This mechanism standardizes the semantic binding process and ensures reproducibility across different HBIM environments. Semantic segmentation methods, semi-automatic modeling of building facades, and multi-layered decomposition inspired by the concept of Level of Development (LoD), structural decomposition are important enriching elements of HBIM datasets.Here, the LoD concept is mentioned solely to illustrate the hierarchical organization that HBIM workflows can support. No formal LoD classification (such as AIA/BIMForum LoD 100–400) was applied in this study, and therefore no LoD levels are assigned to the structural, decorative, or material components of the model. Furthermore, the development of HBIM includes considerations of factors for diagnosing and detecting structural damages, followed by proposing methods for conserving and renovating historic buildings^26^.

**Fig. 3:**
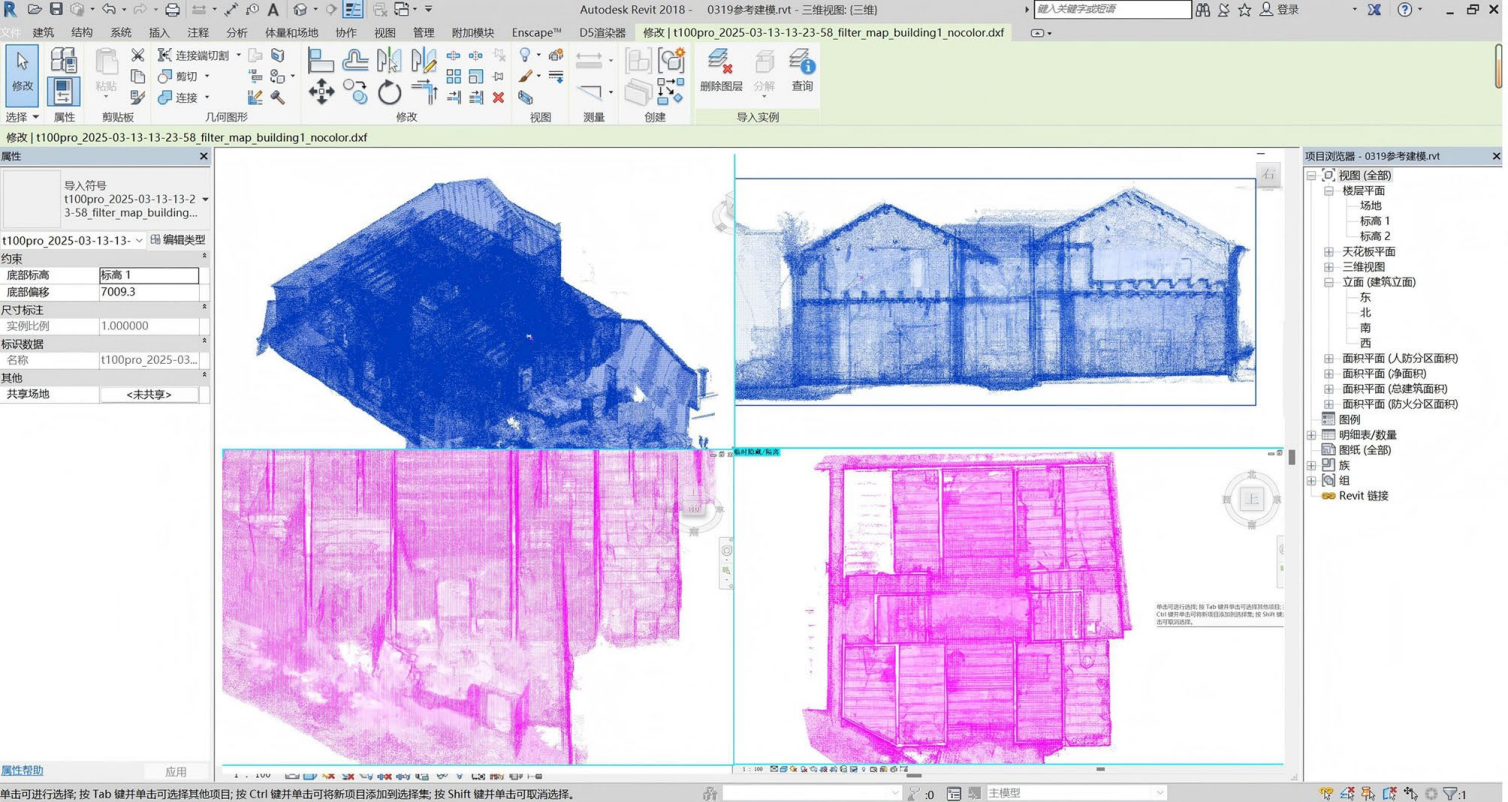
Importing HBIM point cloud data into Autodesk Revit 2018.

**Fig. 4:**
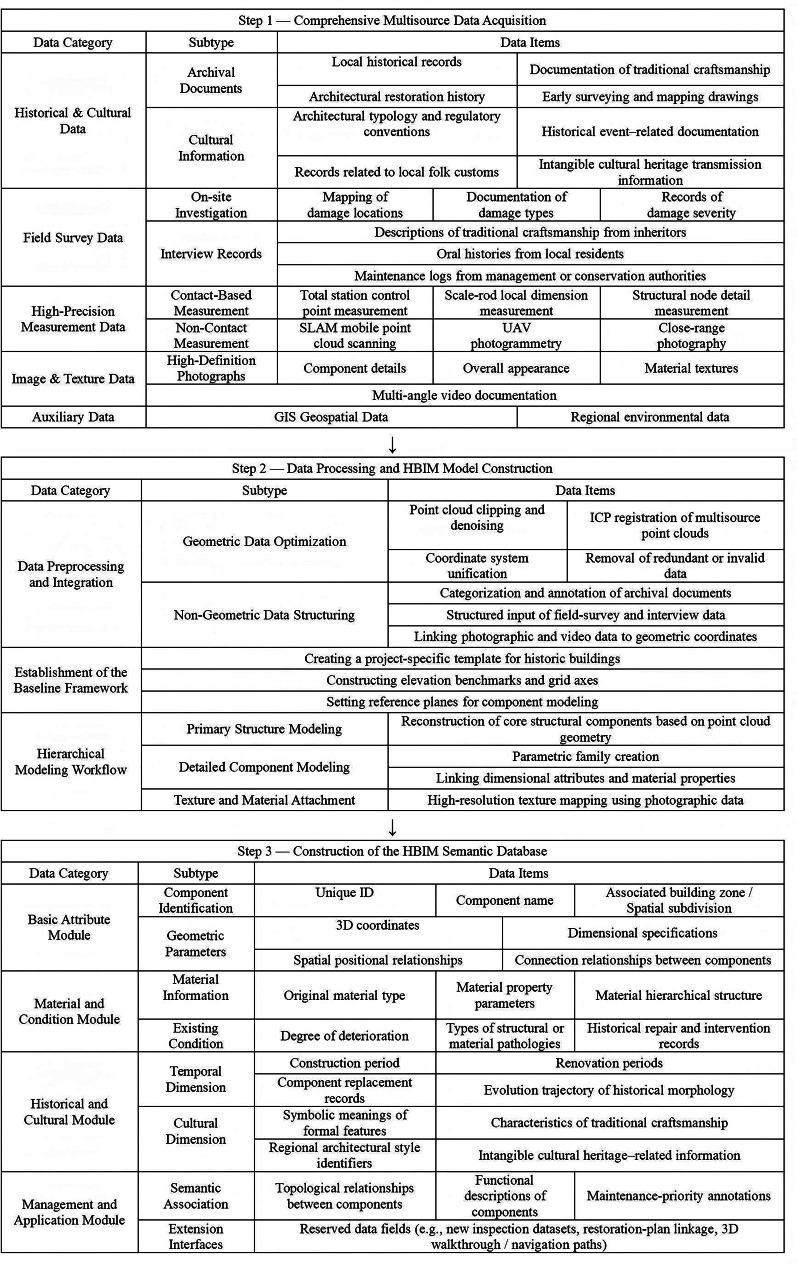
Structured workflow for multisource data acquisition, processing, and HBIM semantic database construction.

During the HBIM construction phase, this study adopted a layered modeling approach, where the overall building structure was first established, followed by the gradual refinement of detailed components such as doors, windows, and decorations. For the Dan Tao’s Former Residence—a structure embodying the characteristics of traditional Qing Dynasty architecture in the Jingchu region—Autodesk Revit 2018 was used to develop an HBIM model integrating multi-disciplinary information^27^. Initially, the raw point cloud data underwent preprocessing steps such as noise filtering, slicing and layering, registration, and redundant trimming^28^, ensuring the integrity and usability of the data. Using Revit’s “Import CAD” function, the .dxf format point cloud was aligned with the 3D coordinate system (Fig. 3); leveraging a parametric component library, detailed modeling of the building structure was achieved by automatically extracting building baselines and core frames through the geometric features of point cloud contours, thereby establishing the spatial layout and vertical dimensions of the “Hui”-shaped courtyard, with subsequent layer-by-layer addition of components like walls, beam frames, and roofs. For features specific to Jingchu vernacular architecture—such as gable walls and coin-shaped vents—precise modeling was conducted using point cloud information, and realistic material properties were assigned to components via texture mapping. Beyond the aforementioned information, the HBIM model displayed door and window styles in the architectural dimension, with each component linked via URL to documents interpreting the symbolic meanings of Qing Dynasty Jingchu architectural features; in the structural dimension, it used “analysis models” to associate the damage status and dimensional parameters of beams and columns. To clarify the semantic workflow, a process diagram(Fig. 4) summarizing the HBIM cultural–semantic association pipeline is provided, illustrating the steps from data organization to parameter binding within Revit. Compared to traditional 3D models, HBIM can output component datasets containing historical information, providing support for heritage restoration material selection, cost estimation, and cultural value assessment. Furthermore, to maintain a coherent semantic structure, all cultural, historical, and symbolic attributes linked through URL parameters follow a unified naming and indexing scheme. Each semantic file is assigned a stable key, and the corresponding Revit parameter stores this key as its reference, ensuring that the cultural narrative remains traceable and can be consistently retrieved regardless of software environment or hardware conditions.

### Knowledge visualization

1. Residential architecture gene recognition method.

This study employed an interdisciplinary approach, primarily utilizing landscape gene analysis and architectural typology methods, integrating fields such as architectural typology, cultural geography, landscape studies, biology, and history. Inspired by gene analysis methods from biology, landscape gene theory constructed recognition indicators to systematically analyze the intrinsic traits, external expressions, and inheritance characteristics of settlement cultural landscapes. By categorizing material forms, using a hierarchical thinking model, and employing visual expressions, the landscape gene theory system was extended and adapted to the study of residential architecture. This approach established a recognition and mapping methodology for identifying the architectural “Genes” of rural dwelling^29^.

First, by referring to Liu Peilin’s principles for identifying landscape genes^30^—namely, the principles of intrinsic uniqueness, extrinsic uniqueness, local uniqueness, and overall dominance—and considering the formation and development patterns of Qing Dynasty vernacular dwellings in the Jingchu region, principles for recognizing residential architecture genes were proposed (Table 1).

**Table 1:** Identification principles of vernacular dwelling genes.

Basic principles	Definition
Intrinsic uniqueness principle	Not possessed by residential houses in other regions in terms of internal origin
Extrinsic uniqueness principle	Not possessed by residential houses in other regions in terms of external form
Local uniqueness principle	Certain local and key elements are not possessed by residential houses in other regions
Overall dominance principle	Residential houses in other regions have similar elements, but this region is more prominent

**Fig. 5:**
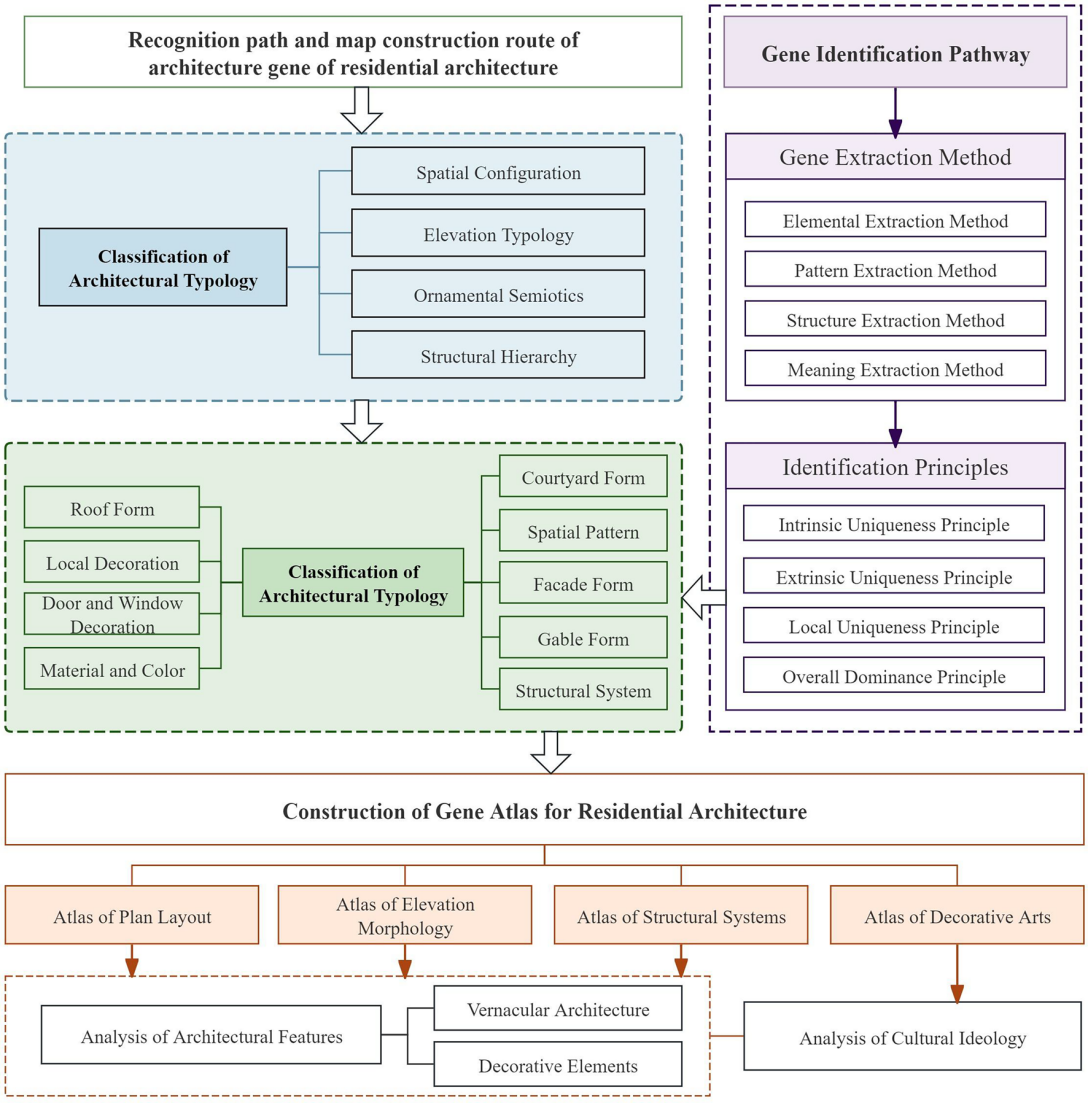
Recognition path and map construction route of architecture gene of residential architecture.

Second, leveraging Shen Xiuying’s methods for landscape gene extraction—which include element extraction, pattern extraction, structure extraction, and meaning extraction^31^—a method was developed for extracting vernacular dwelling genes. Subsequently, based on the hierarchical structure of residential buildings, gene recognition indicators and pathways were established (Fig. 5). Gene recognition and extraction were performed across aspects such as courtyard types, floor plans, building facades, gable wall shapes, roof forms, door and window styles, detailed decorations, materials and colors, and wooden frameworks^32^. Using visual representation techniques, the gene features of Qing Dynasty vernacular dwellings in the Jingchu region were documented, leading to the creation of maps depicting spatial layouts, structural components, and decorative elements of Qing Dynasty residences in the Jingchu region.To increase methodological reproducibility in the identification of intangible architectural genes, the present study applies quantitative criteria to distinguish core cultural features from incidental variations. A cultural feature is defined as a core architectural gene when it meets two quantitative thresholds: (1) an occurrence threshold, whereby the feature appears in at least 70% of comparable historical samples or documented cases; and (2) a significance threshold, whereby the feature is explicitly referenced in authoritative historical texts or constitutes a typologically indispensable structural or decorative rule. These quantitative thresholds provide a standardized basis for gene recognition and ensure consistency across different stages of HBIM semantic annotation. Although the cultural-decoding workflow is primarily qualitative, the HBIM structure is designed to accommodate potential quantitative extensions, including optional metrics such as pattern recurrence counts, spatial regularity checks, and semantic annotation density.

(2) Diagrammatic analysis method.

Diagrammatic analysis is a systematic research method grounded in the ontology of architecture. Its core involves visually deconstructing and reorganizing elements of architectural space, structure, and form to reveal the intrinsic logic and knowledge system underlying design. The method originated in the 1950s with the teaching practices of the “Texas Rangers” school and was further developed by scholars like Bernard Tschumi and Peter Eisenman, creating an analytical framework centered on spatial issues.

During research, original materials such as architectural floor plans, elevations, sections, and isometric drawings are utilized. Techniques including diagram transformation, transparency diagrams (e.g., spatial layering and superposition), and geometric proportion analysis are used to extract subsystem features like spatial boundaries, compositional order, spatial sequences, and construction logic. The analysis follows a systematic checklist addressing dimensions like functional flow, service space hierarchy, structural-space relationships, and environmental interaction.

To verify the authenticity of spatial perception, three-dimensional modeling or abstract models are employed. The theoretical foundation of this method is supported by Colin Rowe’s “Transparency” theory, Bruno Zevi’s spatial diagramming method, and modern systems theory, which conceives architecture as an integrated complex of “Use-Space-Construction-Form.”

First, spatial elements are deconstructed from the original drawings to extract fundamental orders, such as geometric networks and symmetry axes. Next, techniques such as inverting diagrams, overlaying transparent layers, or creating exploded isometric views are used to analyze spatial hierarchical relationships, including public–private gradients and interactions between internal and external transitional spaces. Finally, construction logic is combined with environmental relationships to validate the synergy between form, function, and structure. The research places emphasis on “Outcome-Based Interpretation”, using diagrams to reveal design quality aspects such as spatial innovation, system integration, and the meticulous handling of details^33^.

## Results

In this study, knowledge acquisition—essential for digital preservation—is divided into two key components: data collection and data processing. Multimodal data, such as point cloud data and images, were gathered to thoroughly capture the architectural information of the Dan Tao’s Former Residence. Once processed, this data established a foundation for further in-depth research, and efforts to protect and utilize the relevant architectural heritage information are being systematically conducted.

### Knowledge acquisition

#### Data collection

This study utilizes various devices to collect multimodal data, combining field investigations and literature review to gather historical information. The collected data includes records of the building’s current state, local chronicles, family history documents, historical images, and details about construction dates, functional evolution, spatial layout, family ethics, and Feng Shui patterns, aiming to create a cultural background knowledge base that offers historical evidence for restoration efforts.

**Fig. 6:**
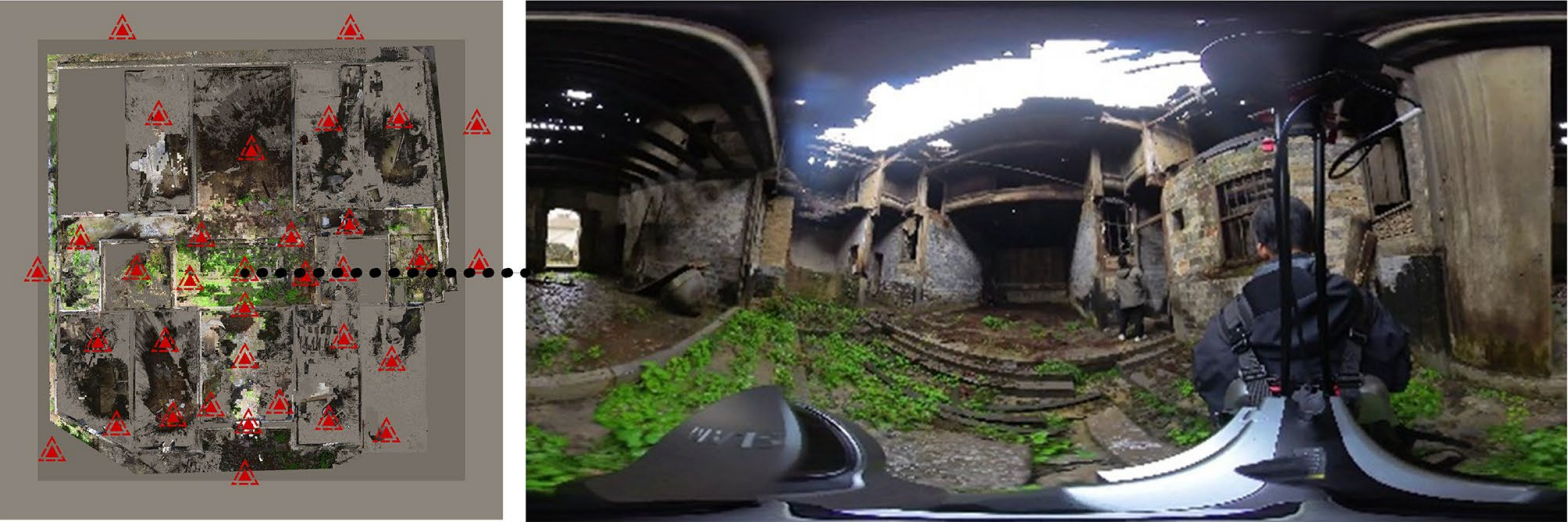
Scanning stops and panoramic photos taken by the GoSLAM T100 Pro RTK.

To support high-precision modeling, 3D laser point cloud data was acquired using the GoSLAM T100Pro RTK 3D laser scanner and other tools. This data captured both the overall and detailed 3D coordinates^34^, RGB color information, and geometric dimensions of the building’s interior and exterior spatial structures, including decorative elements like wood carvings and brick carvings. Specific scanning scene panoramas and the scanning point distributions are illustrated in Fig. 6.

Additionally, the DJI AIR 2 s drone was used to capture images of the building’s macro layout, roof, and surrounding environment, covering vertical and multiple inclined angles to supplement data from scanning blind spots. High-definition images were collected with a Canon camera to document detailed decorations, material textures, and severely damaged part, thereby optimizing the textures of the 3D model. AutoCAD (AutoCAD 2020) was employed to create CAD drawings, extract architectural floor plans, elevations, sections, and geometric parameters, and construct a quantified historical construction database. Specific data is presented in Table 2.

**Table 2:** Description of multi model data.

Data type	Method	Content
Field survey and historical data	On—site investigation, literature collection	Text records: architectural layout, decorative details, historical background Literature materials: local chronicles, family history, some historical images
3D laser point cloud data	GoSLAM T100Pro RTK 3D laser scanner, GoSLAM LidarWorks, CloudCompare	Scanning speed: 640 k points/second Scanning radius: 120 m Coverage: the whole scene inside and outside the residential house (2 courtyards, 3 sky wells, 12 rooms) Relative precision: 1 cm (in line with CH/Z3017—2015 standard) Coordinate system: WGS84, CGCS2000
UAV aerial photography data	DJI AIR 2 s	Aerial photos: 31 (including vertical and multi—tilt angles) Flight altitude: 50 m Overlap rate: 75% along the course, 65% lateral Image resolution: 5472 × 3648 (3:2), 5472 × 3078 (16:9) Viewing angle: 88° (equivalent focal length 22 mm)
High—definition image data	Canon EOSR6 MarkII	Total number of photos: 186 Resolution: 24.2MP Post—processing: 186 images are color—corrected and denoised by Lightroom, generating PNG format texture maps
CAD drawing data	AutoCAD software	Drawing types: plan, elevation, section Collection content: courtyard layout, room size, component specifications Precision: millimeter—level (extracted based on point cloud data)

#### Data processing

This study employs a systematic data processing approach to optimize laser point cloud and image data, improving data quality and modeling accuracy to support the digital preservation of cultural heritage. During the point cloud processing phase, GoSLAM LidarWorks software was employed to remove redundant points and regulate density through resampling algorithms. Three-dimensional clipping was used to eliminate environmental noise, isolating the point cloud of the target building. Layered registration was applied to fuse multimodal point clouds, extracting key architectural contours through contour feature slicing, and enhancing surface texture via texture mapping.In this study, multimodal point cloud registration was performed using the Iterative Closest Point (ICP) algorithm integrated within the GoSLAM LiDAR workflow. The SLAM system combines LiDAR and IMU sensors, enabling high-accuracy localization and scene transition without requiring GNSS assistance. As a result, the registration process does not rely on ground control points (GCPs) or check points, while ensuring consistent coordinate alignment across datasets.

In the image processing phase, techniques for generating floor layouts and extracting façade features were used to analyze architectural structural parameters^35^. Adobe Lightroom software facilitated the correction of color space, dynamic range optimization, and noise reduction across 186 images^36^. These images were exported as high-definition PNG texture maps that comply with industry standards, ensuring a realistic portrayal of materials on the 3D model.

**Fig. 7:**
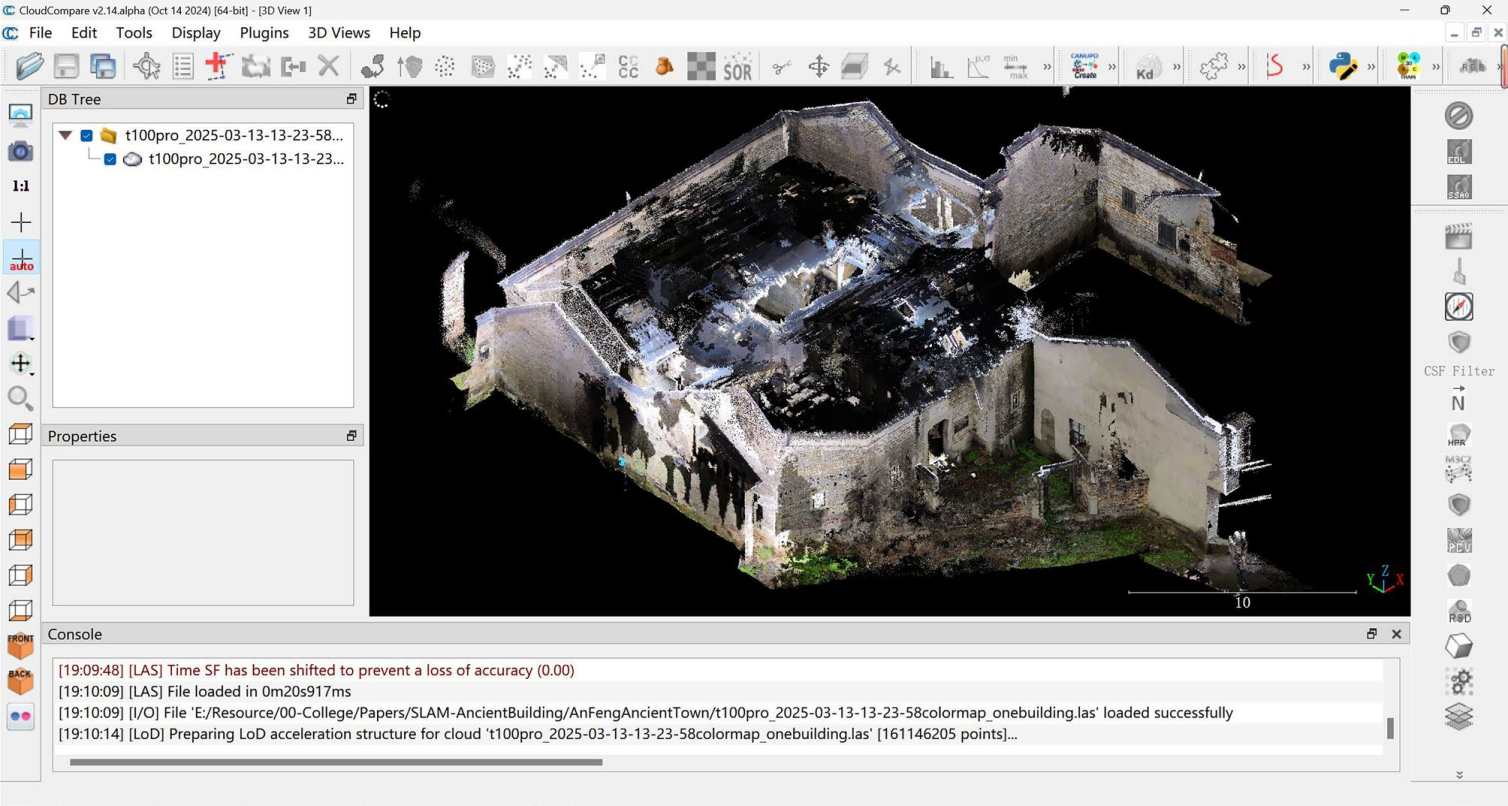
Laser point cloud (LPC) in cloudcompare software.

For sections of the building with severe damage, the CloudCompare (V2.14. alpha) platform integrated with SketchUp 2022 and Undet plugin functions to perform reverse modeling of the point cloud and implement parametric repair techniques. This was complemented by layered registration, contour feature slicing, and texture mapping optimization, completing the digital reconstruction of severely damaged structures, as depicted in Fig. 7. This robust processing workflow significantly enhances data quality and modeling accuracy.

In this study, a total of 186 images taken were classified as shown in Table 3.

**Table 3:** Photo categorization directory.

Overall category	Shooting device	Sub-category	Specific detail
All Images	Camera	Windows	Window lattice patterns
			Window decoration carvings
		Doors	Door lintel
			Door post
			Door hinge
		Walls	Brick pattern laying method
			Cornerstone
			Plinth
	Drone	Low-altitude details	Roof tiling
			Plinth
		High-altitude overlook	Building complex layout
			Surrounding environment

#### HBIM reconstruction

**Fig. 8:**
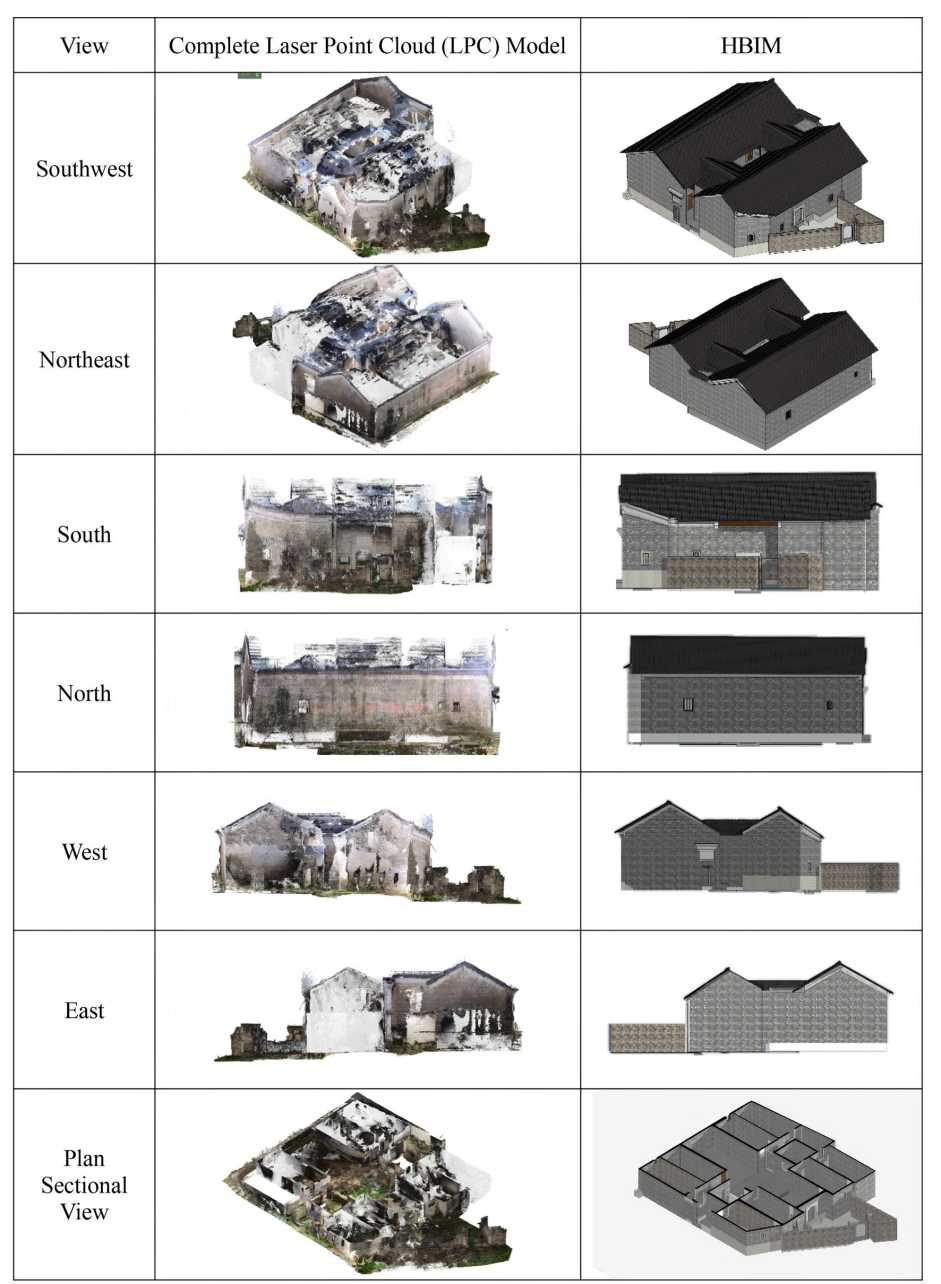
Comparison between raw point cloud and HBIM of the entire building.

**Fig. 9:**
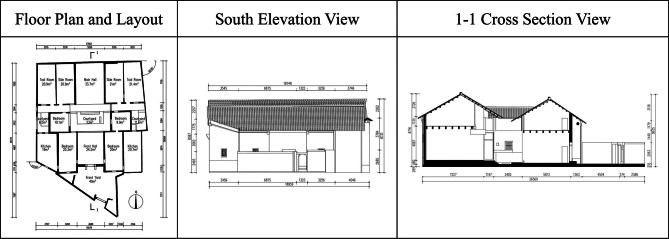
Line drawings.

**Fig. 10:**
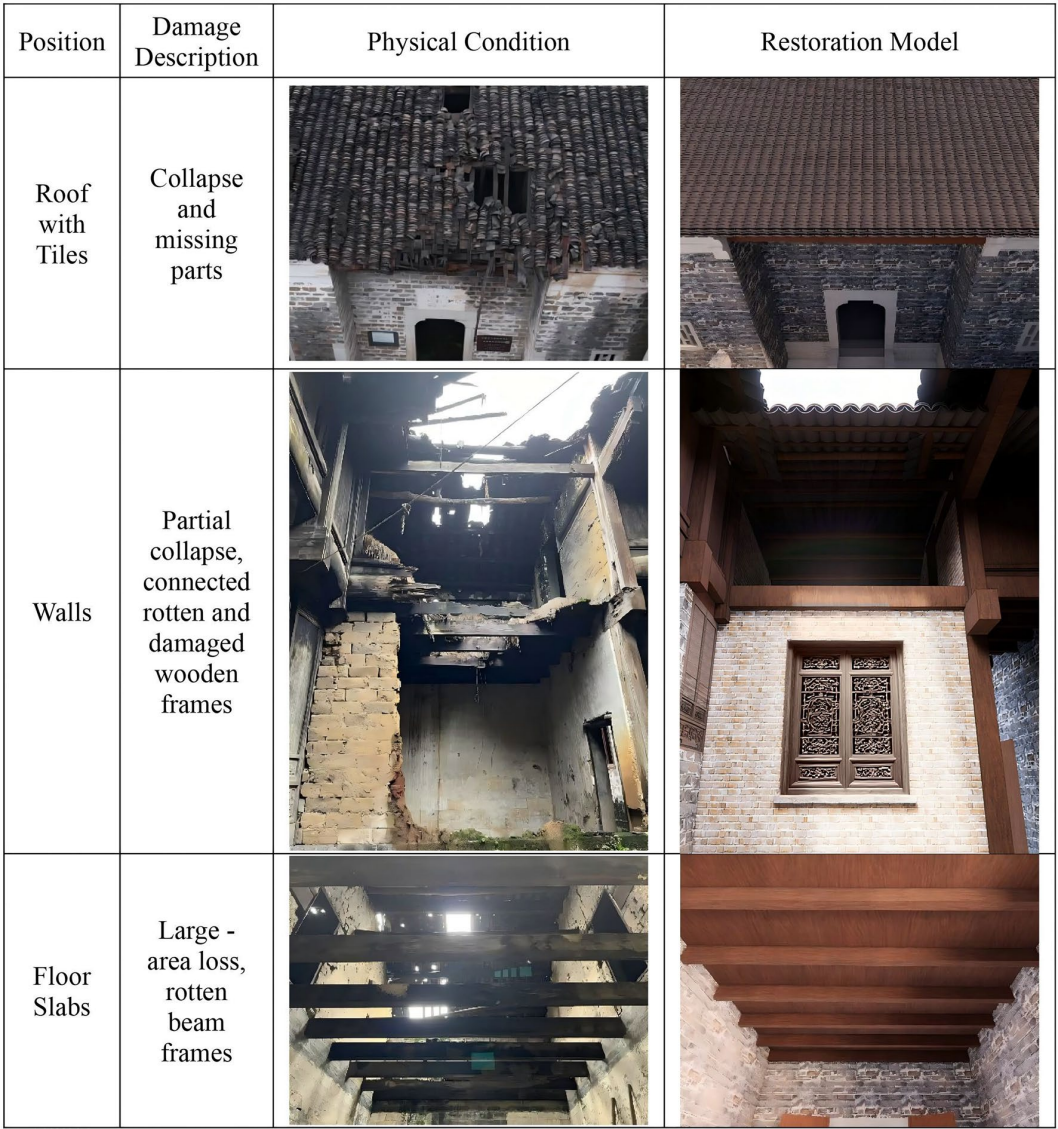
Comparison between physical damage and restoration model of interior typical structures.

This study employed multimodal data fusion technology to accurately reconstruct the HBIM of the Dan Tao’s Former Residence. Initially, UAV oblique photography was used to repair spatial topology in areas where point cloud data was missing, particularly on both sides of the roof ridge. This provided multimodal data references to compensate for missing point cloud data. In CloudCompare software, missing roof sections were fitted and repaired, and redundant areas were calibrated and cropped to establish an accurate point cloud data foundation for HBIM reconstruction. (Fig. 8) The technical drawings, including floor plans, elevations, and sections generated in AutoCAD in Fig. 9, provided precise measurements. This technical system effectively addressed the issue of missing point cloud data in areas impacted by damaged tiles, rotten beams, broken walls and missing floor slabs in the Dan Tao’s Former Residence. Figure 10 illustrated model reconstruction and repair of corresponding part.

To enhance methodological transparency, a post-classification assessment was carried out to distinguish components reliably derived from complete point-cloud observations from those reconstructed through analogy-based inference in areas affected by data loss. Each inferred or partially inferred element was assigned a descriptive engineering confidence level (High / Moderate / Low), determined by point-cloud completeness, geometric continuity, and structural comparability. This clarification provides a concise and engineering-oriented indication of reconstruction uncertainty, with representative components summarized in Supplementary S2.

**Fig. 11:**
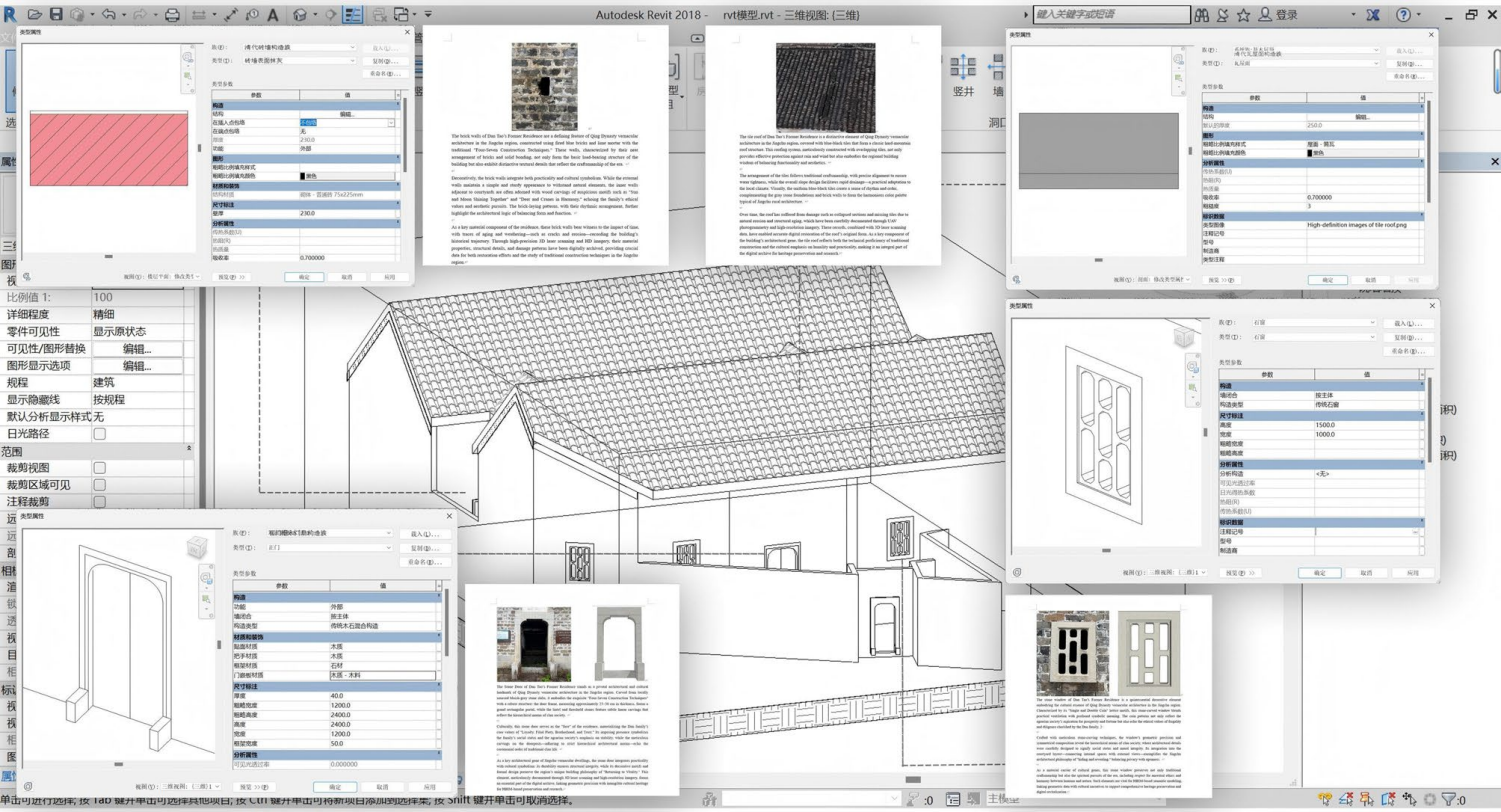
The association between each component of HBIM and information.

As shown in Fig. 11, the HBIM model formed a multi-dimensional and precise archive, which can clearly and conveniently associate and present the geometric forms and historical-cultural information of building components, thus establishing the architectural knowledge system and cultural map of the Dan Tao’s Former Residence.

**Fig. 12:**
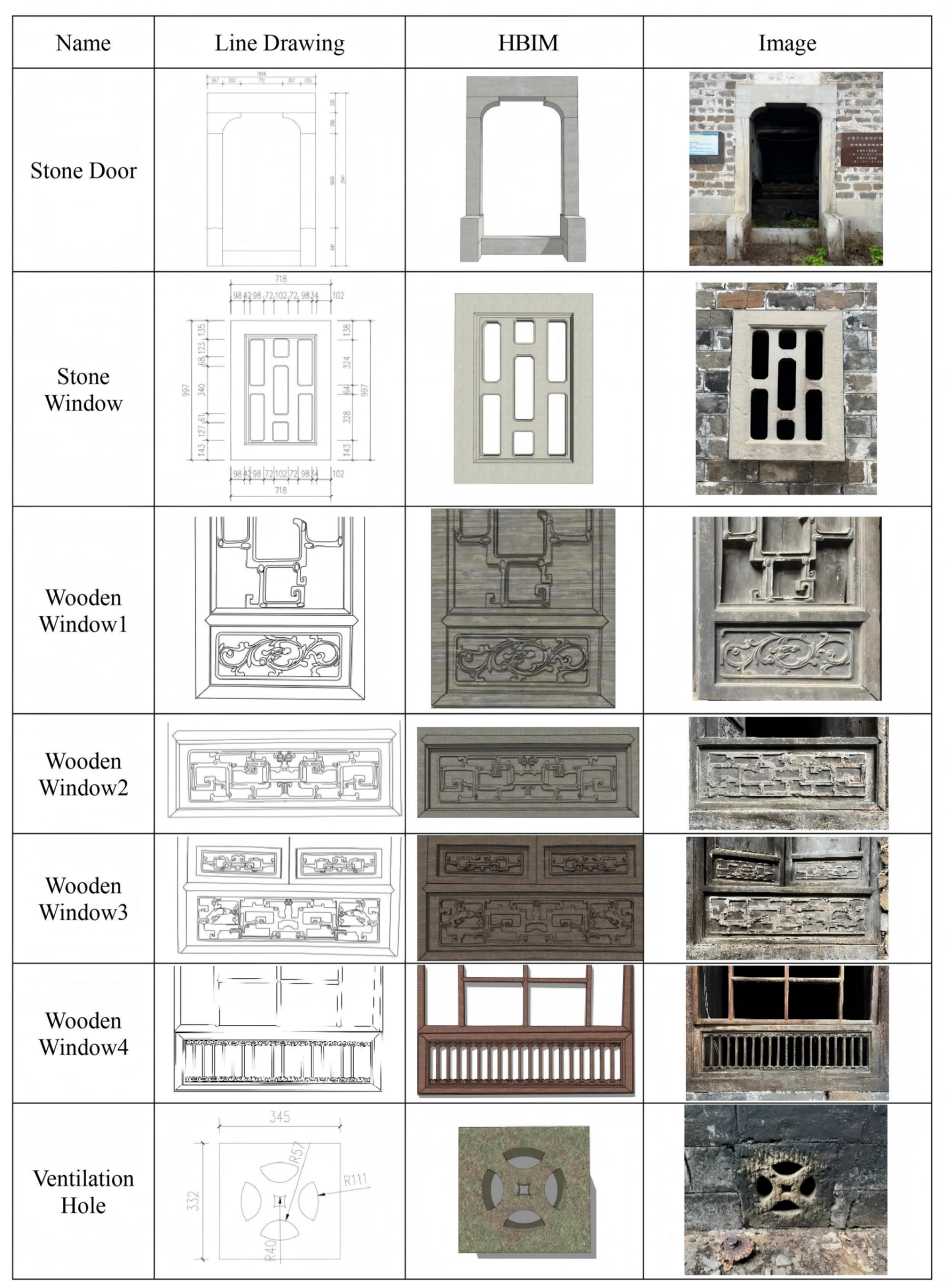
Comparison of line drawings, HBIM and images of decorative component.

This study extracted both the macroscopic dimensions and millimeter-level detailed component measurements of the Dan Tao’s Former Residence utilizing high-precision point cloud data. Typical architectural component Line drawings, 3D models, and image comparisons presented in Fig. 12 visually revealed the multidimensional data relationships of components such as stone doors, stone windows, and wooden windows. This offered a visual reference for accurately restoring architectural details.

#### Accuracy verification results of HBIM component restoration

To evaluate the geometric fidelity of the restored HBIM components, ICP-based registration and cloud-to-cloud deviation analysis were performed in CloudCompare for five representative component categories, including stone windows, wooden windows, stone doors, and beam frames. The quantitative results are summarized in Table 4, and the detailed processing workflow is provided in the SupplementaryS1.

**Table 4:** Accuracy verification of component ICP registration.

Component type	Macro size (mm)	Registration point count	Root mean square error (RMS/m)	Absolute Error range (m)	Relative error (%)	Registration status
Stone window	1000	50,000	0.0193	0.003–0.250	1.93	100% Overlap
Wooden window 01	1500	50,000	0.0059	0.002–0.087	0.39	100% Overlap
Wooden window 02	1500	49,818	0.0035	0.001–0.064	0.23	100% Overlap
Stone door	2500	50,000	0.0271	0.004–0.112	1.08	100% Overlap
Beam frame	5500	50,000	0.0633	0.009–0.305	1.15	100% Overlap

All components achieved high-quality alignment, with RMS values ranging from 0.0035 m to 0.0633 m. Wooden Window 02 exhibited the highest accuracy (RMS = 0.0035 m), followed by Wooden Window 01 (0.0059 m). The stone window (0.0193 m), stone door (0.0271 m), and beam frame (0.0633 m) displayed proportionally larger RMS values consistent with their macro-scale dimensions.

Relative errors for all five components remained below 1.93%, well within the commonly accepted 3% tolerance for digital heritage restoration. Wooden window components showed exceptionally low relative errors (0.23–0.39%). Although the beam frame exhibited the highest absolute deviation (0.0633 m), its relative error remained low (1.15%), reflecting the expected correlation between component size, point-cloud density, and accumulated deviation.

**Fig. 13:**
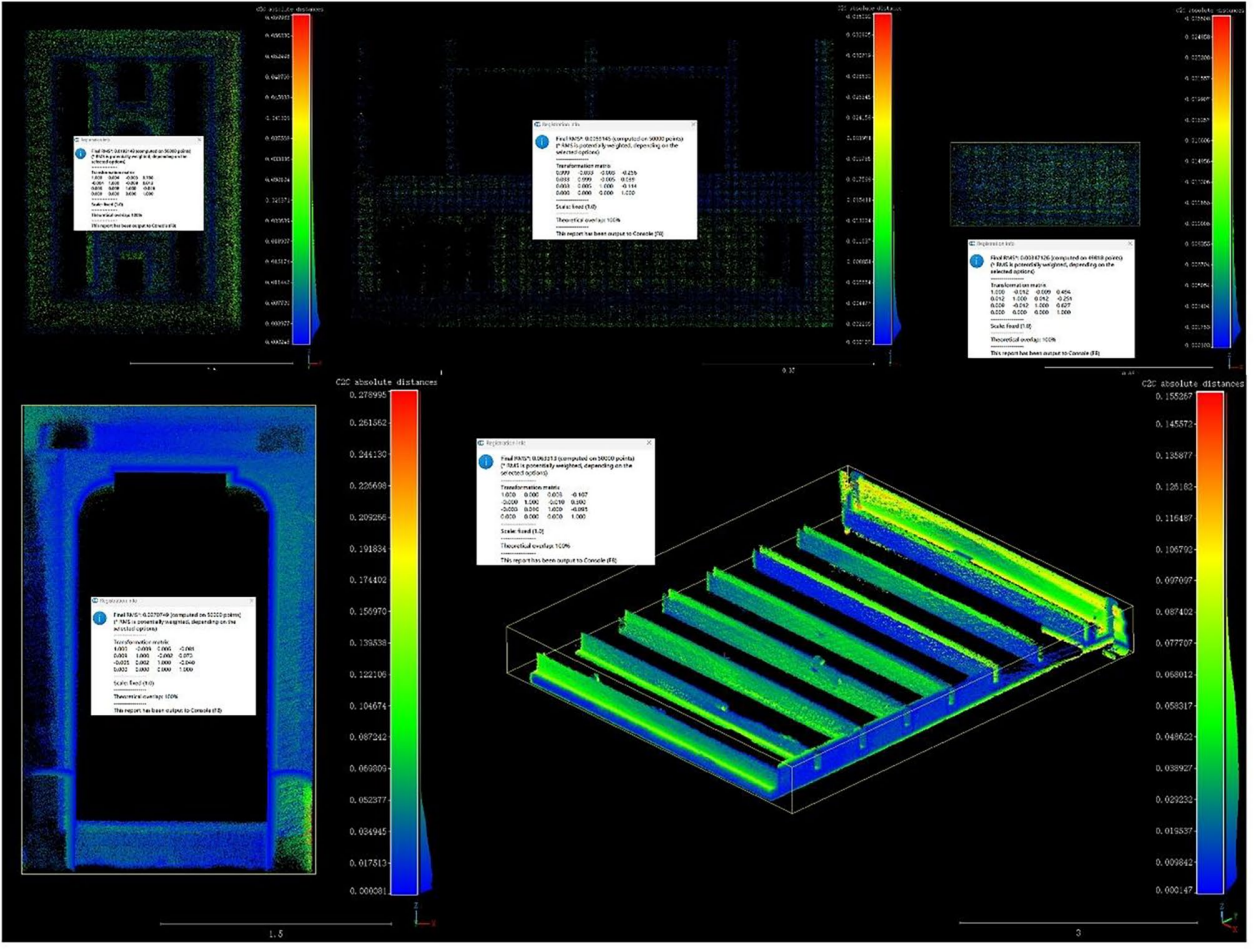
Cloud-to-cloud deviation heatmaps for five representative HBIM components.

Data reliability was ensured by a minimum of 49,818 registration points per component, and CloudCompare reported 100% theoretical overlap for all cases, confirming complete geometric correspondence without missing regions. Deviation-map visualization further showed predominantly low-error distributions, indicating strong consistency between HBIM reconstructions and reference point clouds. Representative deviation heatmaps and RMS value distributions for each component (Fig. 13) further illustrate the spatial characteristics of the errors, highlighting the predominance of low-deviation regions and the localized nature of higher discrepancies.

Collectively, these results demonstrate that the reconstructed HBIM components achieve the level of morphological fidelity and detail preservation required for Qing-Dynasty architectural heritage, providing a robust geometric foundation for subsequent semantic annotation, cultural interpretation, and digital presentation.

#### Knowledge visualization and construction of knowledge graphs

This study employed the pyramid principle to reconstruct the knowledge structure of the Dan Tao’s Former Residence, creating a mind map using Xmind software. The map utilized data from two main knowledge visualization databases: the foundational knowledge visualization database and the material database, marked in red and blue respectively, to emphasize the logical relationships between branches, clearly identifying connections between each knowledge point.

The mind map centered on the Dan Tao’s Former Residence, employing vernacular dwelling gene recognition for definition. This recognized both material and immaterial architectural genes. Material architectural genes encompassed physical features of the residence, divided into overall features and detailed decorations. Overall features included aspects such as building form, roof shape, gable style, door and window designs, and architectural textures. Detailed decorations covered decoration locations, methods, and themes.

Immaterial architectural genes focused on cultural characteristics, expressed through regional cultural influences. From field surveys and literature in Anfeng Village, Chibi City, these cultural traits were categorized into themes such as the legacy of agriculture and education, loyalty, filial piety, integrity, and themes related to wealth and prosperity.

**Fig. 14:**
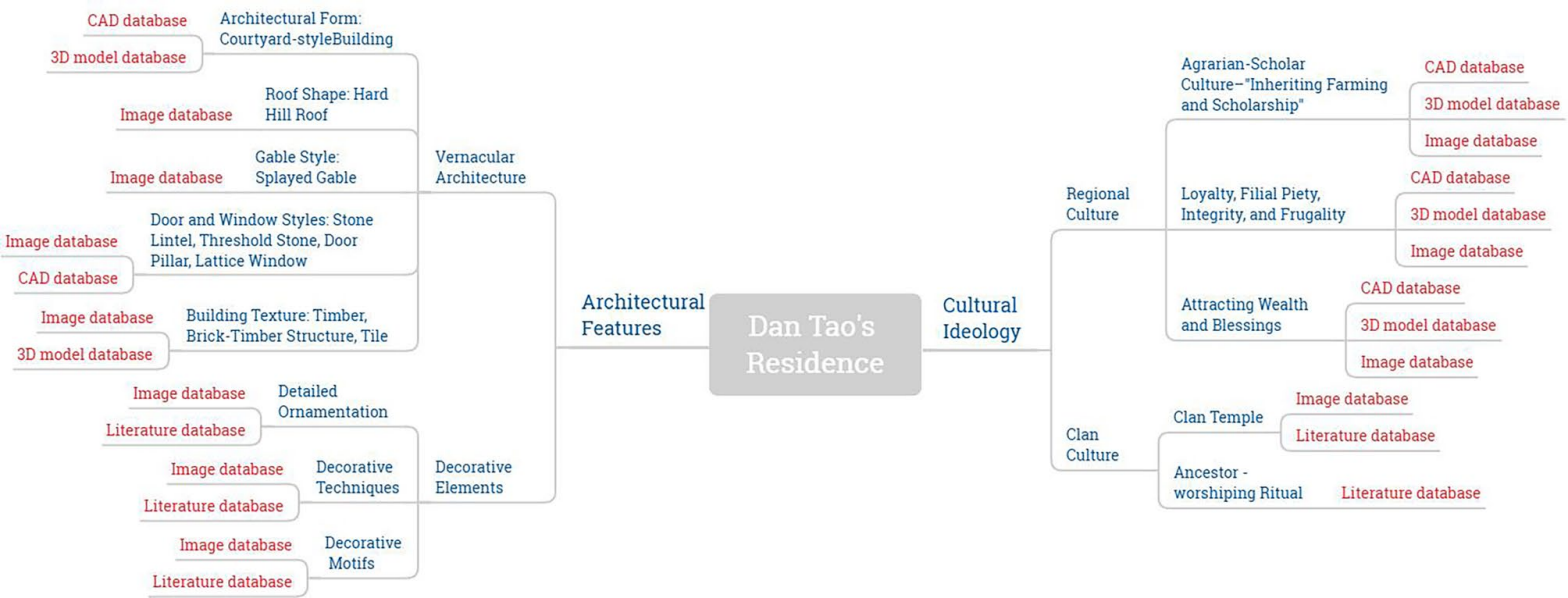
Architectural gene identification and typology.

These classifications were organized into primary branches, with database components as secondary branches. Multidimensional thinking led to the derivation of third-level branches, encapsulating attribute information within the tree-structured mind map. The visual analysis of typical vernacular architectural features, extracted from the restoration model’s architectural details, was illustrated in Fig. 14.

To ensure methodological rigor, quantitative validation was conducted by comparing SLAM-derived point clouds against terrestrial laser scanning (TLS) benchmarks and manual ground-truth measurements. Root-mean-square error (RMSE) values were calculated for both global alignment and local decorative components. This analysis provides objective metrics for assessing data accuracy and ensures the reliability of the proposed multimodal workflow.

## Discussion

### Analysis of architectural features

1. Vernacular architecture.

**Fig. 15:**
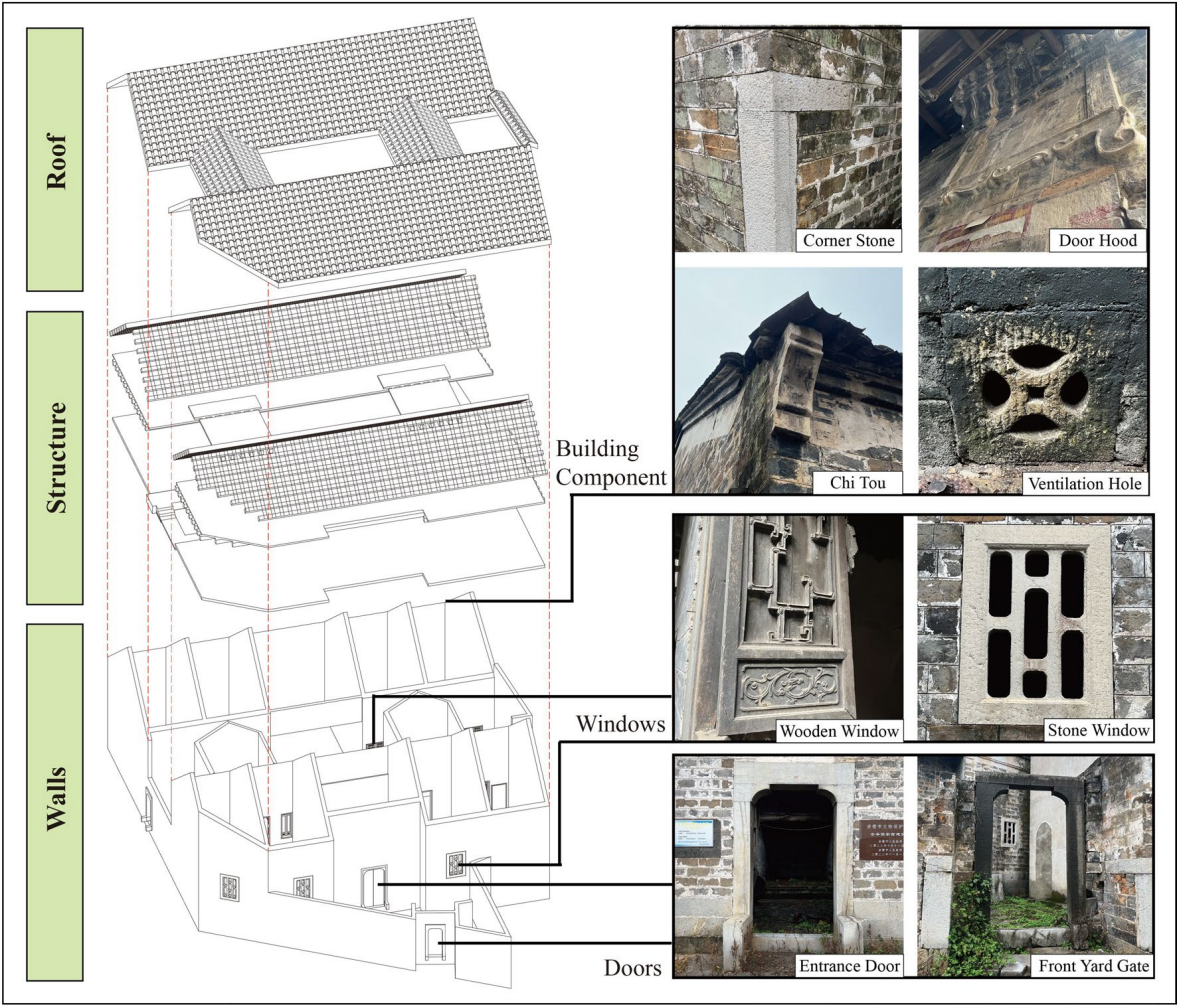
Diagram of vernacular architectural features of Dan Tao’s former residence.

The Dan Tao’s Former Residence, as analyzed through this restoration model, exhibited distinct vernacular architectural characteristics. It featured a courtyard-style layout with a hard-mountain roof and gable walls. Architectural elements of the Dan Tao’s Former Residence included traditional features like stone lintels, threshold stones, door columns, and lattice windows. The building materials employed included a blend of wood, brickwood, and tiles, representing the typical construction techniques and material choices of the Qing Dynasty’s Chibi region. This combination ensured structural stability while showcasing the aesthetic preferences of rural architecture from that era and region. The specific vernacular architectural features compiled in this study were further illustrated in Fig. 15.

(2) Decorative elements.

This study employed a systematic, multidimensional approach to analyze the detailed decoration system of the Dan Tao’s Former Residence. Utilizing high-resolution imagery and 3D laser point cloud scanning, the geometric characteristics and carving depth variations of wood-carved window lattice patterns were accurately documented. For the restoration of construction techniques, traditional carving methods like “Layered Scraping” and “Carved Layering” were systematically identified through microscopic image analysis and historical document research.

**Fig. 16:**
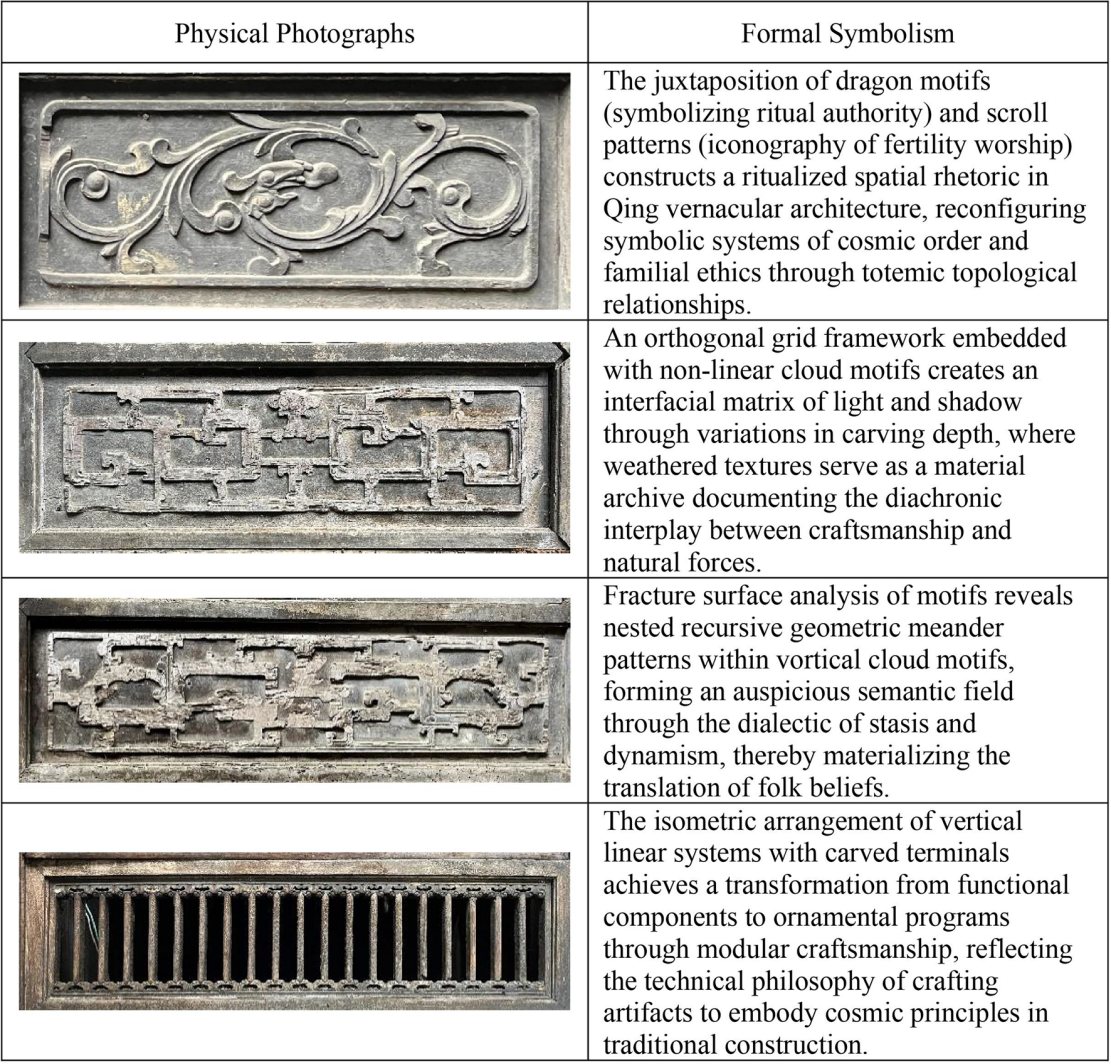
Extraction of ornamentation elements and analysis of morphological characteristics.

In terms of cultural symbol decoding, a decorative motif database was created using semiotic theory to elucidate the spatial narrative reconstructed by the juxtaposition of dragon and scroll patterns, and the philosophical expressions of order and flow achieved through the combination of orthogonal grids and cloud motifs. This study unveiled the deep coupling mechanism between technical practices and cultural metaphors in the Jingchu vernacular decoration system through three main pathways: morphological analysis, craft tracing, and semantic interpretation. Detailed extraction and analysis of decorative elements were provided in Fig. 16.

### Analysis of cultural ideology

This study built a cultural component database for the Dan Tao’s Former Residence, focusing on cultural ideology. By using point cloud capture, photographic collection, and model reconstruction, it systematically organized architectural elements like stone doors, stone windows, wooden windows, copper coin-style ventilation openings, and wooden window carvings. These components were rich in cultural meaning.

For example, the stone door, with its imposing and grand presence, served as the “Face” of the building, embodying the family philosophy of “Legacy through Agriculture and Education.” It symbolized the agrarian society’s focus on stable foundations while subtly expressing hoped for knowledge continuity. The structured design of the stone windows mirrored the pursuit of “Integrity,” rooted in the traditional virtues of “Loyalty, Filial Piety, Righteousness, and Thrift,” and highlighted moral standards through meticulous design.

The copper coin-style ventilation openings ingeniously combined practical function with the desire for “Wealth and Prosperity,” with the coin’s form symbolizing aspirations for a prosperous life. The elaborate wood carvings on the windows, whether they exhibit symmetry or simple ancient charm, reflected the virtues of “Righteousness” and “Thrift,” as well as the aesthetic refinement associated with the agricultural and educational ethos.

Through preserving these architectural details and exploring their deeper implications, the study provided insights into the residence’s transmission of “Agricultural and Educational” culture, the moral infusion of “Loyalty, Filial Piety, Righteousness, and Thrift,” and the auspicious wishes for “Wealth and Prosperity.” Concurrently, it established a cultural database for Qing Dynasty Jingchu vernacular dwellings, facilitating a rich dialogue between the tangible architectural forms and the cultural ideologies they express.

### Limitations and future research directions

Although this study effectively employs multimodal data fusion for digital restoration, certain limitations remain that can be addressed in future work. One challenge is the data resolution and coverage, particularly in areas with severe structural damage, such as collapsed roofs and missing floors. While the SLAM 3D laser scanning and UAV photogrammetry methods used in this study provide high precision for larger features, their resolution (approximately 1 cm) is insufficient for detailed decorative elements like carvings. To improve accuracy, future studies could explore higher-density scanning technologies, such as Terrestrial Laser Scanning (TLS), or employ data interpolation techniques to enhance coverage and fill in data gaps.

In addition to improving data density, incorporating Virtual Reality (VR) and Augmented Reality (AR) could offer interactive ways to experience and explore digital models of heritage sites. These immersive technologies would allow users to interact with architectural details and cultural symbols in a more intuitive and engaging way.

Another promising direction is the integration of AI-driven pattern recognition to automate the identification and classification of decorative motifs and cultural symbols. By utilizing Convolutional Neural Networks (CNNs), AI could accelerate the process of recognizing intricate carvings, improving both efficiency and accuracy in cultural analysis.

Due to the limited number of available samples, this study adopts a qualitative cultural–semantic interpretation while deferring the construction of a full quantitative index system to future work; the framework is demonstrated through a structurally complete and typologically representative Jingchu dwelling for methodological clarity, yet its modular structure supports extension to diverse dwelling types and preservation conditions.

## Conclusion

This study focuses on the characteristics of Qing Dynasty dwellings in the Jingchu region, employing digital modeling technologies like SLAM laser scanning along with multimodal data collection, processing techniques, and analogy-based inference to devise restoration plans. This approach led to the successful high-precision HIBM of the Dan Tao’s Former Residence, with a systematic analysis of its architectural and cultural features via a recognition system. Key conclusions are summarized as follows:

1. Comprehensive framework development: the study integrated multimodal data fusion, HBIM, and knowledge visualization technologies to create a comprehensive framework for the digital preservation and revitalization of Qing Dynasty vernacular dwellings in Jingchu. By employing SLAM laser scanning, UAV photogrammetry, and high-definition imagery, it achieved high-precision digital recordings of detailed aspects like spatial layout and decorative elements, even for severely damaged structures.
2. High-precision reconstruction: by deeply integrating multimodal data with point cloud processing, this study overcame traditional challenges such as low point cloud density, missing data from blind spots, and limitations in detail precision. This allowed for accurate HBIM reconstruction with damaged tiles, broken beams, and missing floors, providing a strong data foundation for virtual restoration and structural analysis of historical buildings.
3. Cultural feature analysis: using the Dan Tao’s Former Residence in Anfeng Village, Chibi City, as a case study, the research established a cultural feature recognition system through vernacular dwelling gene identification and diagrammatic analysis. A visual knowledge map was created to systematically display the unique spatial construction logic, decorative motifs, and cultural nuances of Jingchu vernacular buildings. This framework addresses data gaps and explores cultural connotations in heritage preservation, offering interactive digital archives for endangered architectural heritage and converting physical heritage into digital assets.

By integrating digital technologies with cultural semantics, this study enriches the theoretical and methodological framework for architectural heritage preservation. It establishes a complete technical chain of “Data Collection—Model Reconstruction—Cultural Decoding,” presenting a new paradigm for the revitalization and transmission of tangible cultural heritage. Practically, high-precision HBIM and cultural feature maps can inform restoration plans, educational programs, and traditional village tourism development, transforming cultural resources into sustainable development drivers. Additionally, this research aligns with China’s cultural relics digital preservation policies, demonstrating the feasibility of technology-driven methods in uncovering deep cultural values of architectural heritage, and offers a replicable path for protecting and revitalizing traditional buildings in similar regions.

## Supplementary Information

Below is the link to the electronic supplementary material.

Supplementary Material 1

## Data Availability

The datasets generated and/or analyzed during the current study are available from the corresponding author upon reasonable request. Representative point-cloud data, HBIM models, and knowledge-graph assets will be deposited in an open repository (e.g., Zenodo) upon acceptance, in compliance with Scientific Reports data-sharing policies.
