# Supplementary material for: Digital restoration and feature recognition of a Qing-Dynasty vernacular dwelling based on multimodal data fusion
Source: Sci Rep. 2025 Dec 13;16:1844. doi: 10.1038/s41598-025-31544-7 (PMC12804802; doi:10.1038/s41598-025-31544-7)
Supplement: Supplementary file 1 — Supplementary Material 1 [file 41598_2025_31544_MOESM1_ESM.docx]

# Supplementary Material

## S1. Technical Workflow for Accuracy Verification of Digital Restoration of HBIM Components

Description: This study used CloudCompare to implement geometric-accuracy verification between HBIM restored models and real laser-scanning point clouds. The workflow is as follows:

1. Model Extraction and Format Conversion

The target components (stone windows, wooden windows, stone doors, beam frames, etc.) were individually selected in SketchUp (SU). Using File → Export → 3D Model, the models were exported in .obj format. Decorative details were preserved, while redundant faces were simplified to avoid computational delays during subsequent analysis.

2. Preprocessing of Raw Point Clouds

The building-wide .las point cloud data were imported, and CloudCompare’s clipping tool was used to retain only the local region corresponding to each component, thereby reducing irrelevant data. Noise was removed using the Clean-SOR Filter, ensuring improved data reliability.

3. Generation of Model Point Clouds

The exported .obj component models were imported into CloudCompare. Using Mesh → Sample Points / Points Sampling on Mesh, the meshes were converted into point-cloud data. The sampling density was aligned with the raw scan data to support subsequent registration and error computation (Fig. S1).

**
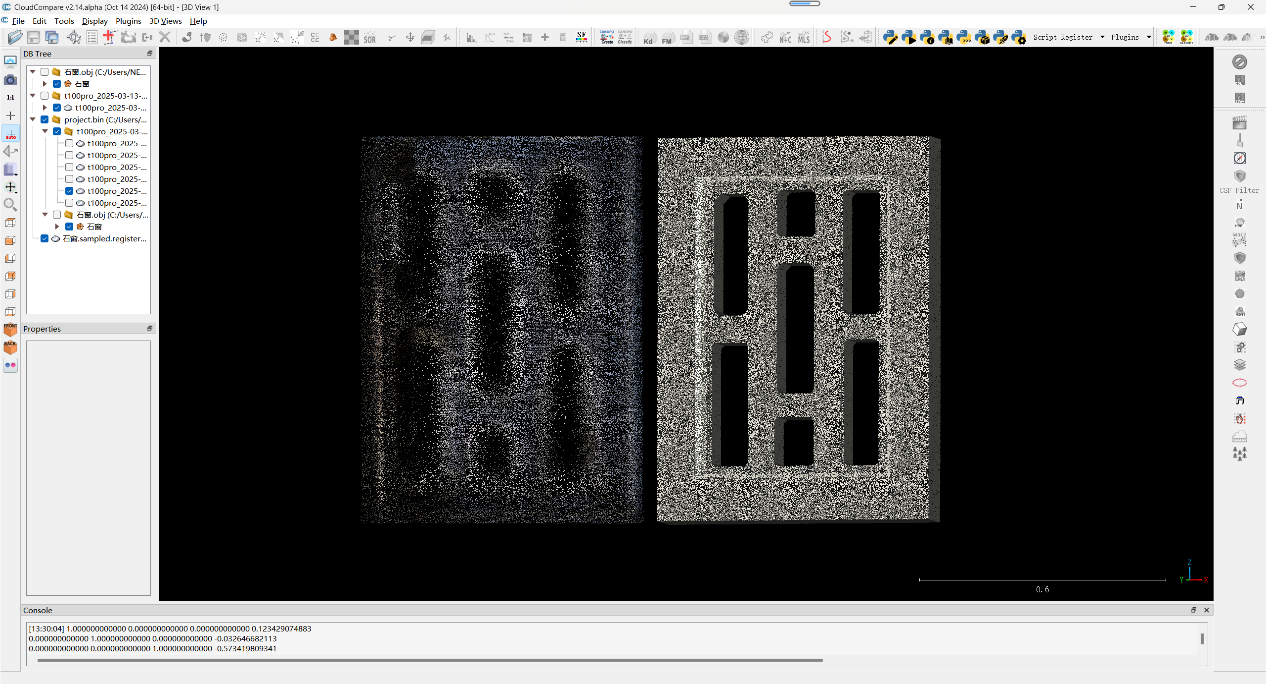
**

Fig. S1. Mesh converted to sampled point cloud.

4. ICP Registration Alignment

The preprocessed raw point cloud and the model-derived point cloud were simultaneously selected. ICP registration was performed via Tools → Registration → Fine Registration (ICP), enabling precise coordinate alignment by enabling Align meshes to points. The initial RMS value was output after registration (Fig. S2).


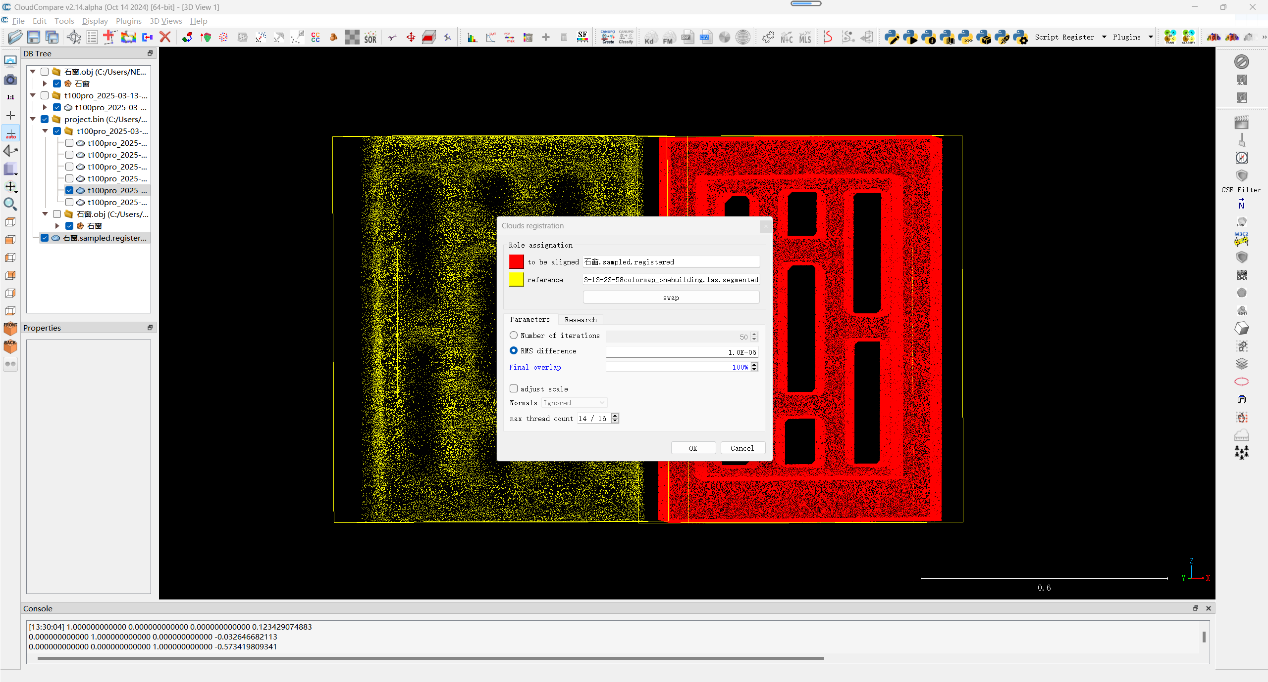


Fig. S2. ICP alignment of model and scan.

5. Quantitative Error Computation

After registration, Cloud-to-Cloud Distance was used to compute the spatial deviation between the two point clouds, generating global RMS, mean deviation, maximum deviation, and other quantitative indicators. All accuracy data were fully documented (Fig. S3).


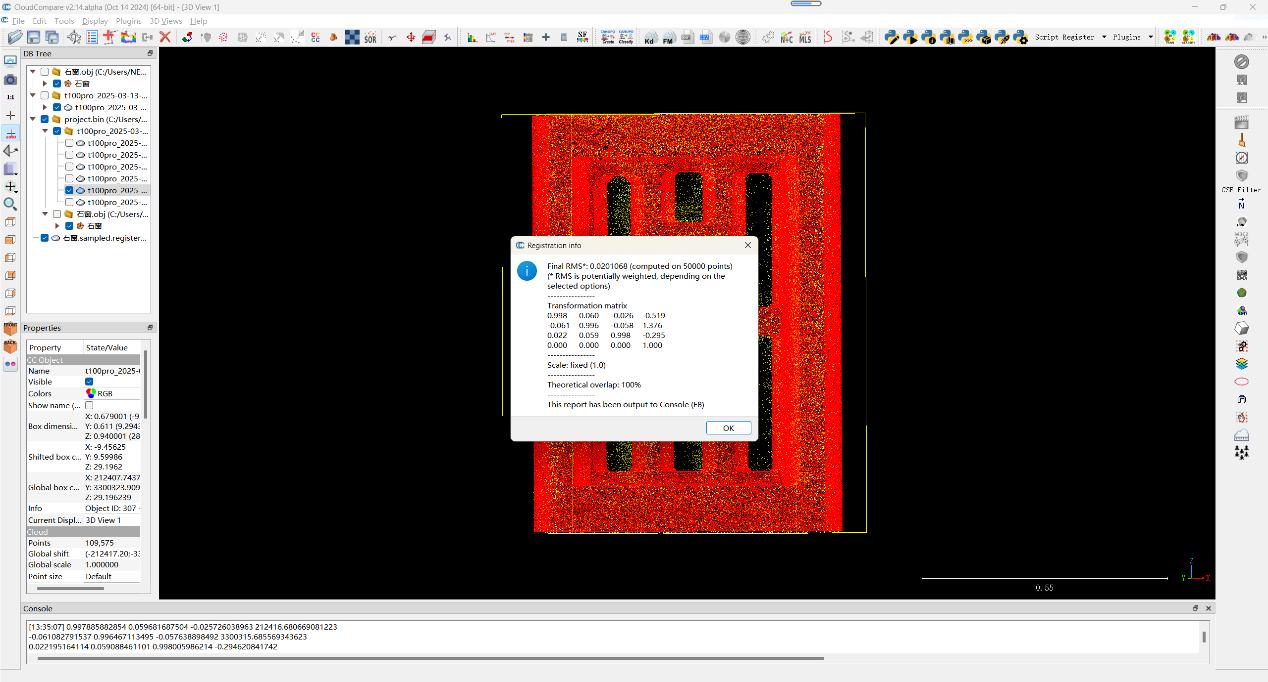


Fig. S3. Cloud-to-cloud geometric deviation analysis.

6. Visual Representation of Error Distribution

CloudCompare’s color-mapping function was used to create error heatmaps (Fig. S4)with a blue → green → yellow → red gradient, where blue indicates minimal error and red represents maximum deviation. The heatmaps revealed the overall and local error distributions. A predominantly blue–green map signified high restoration accuracy.


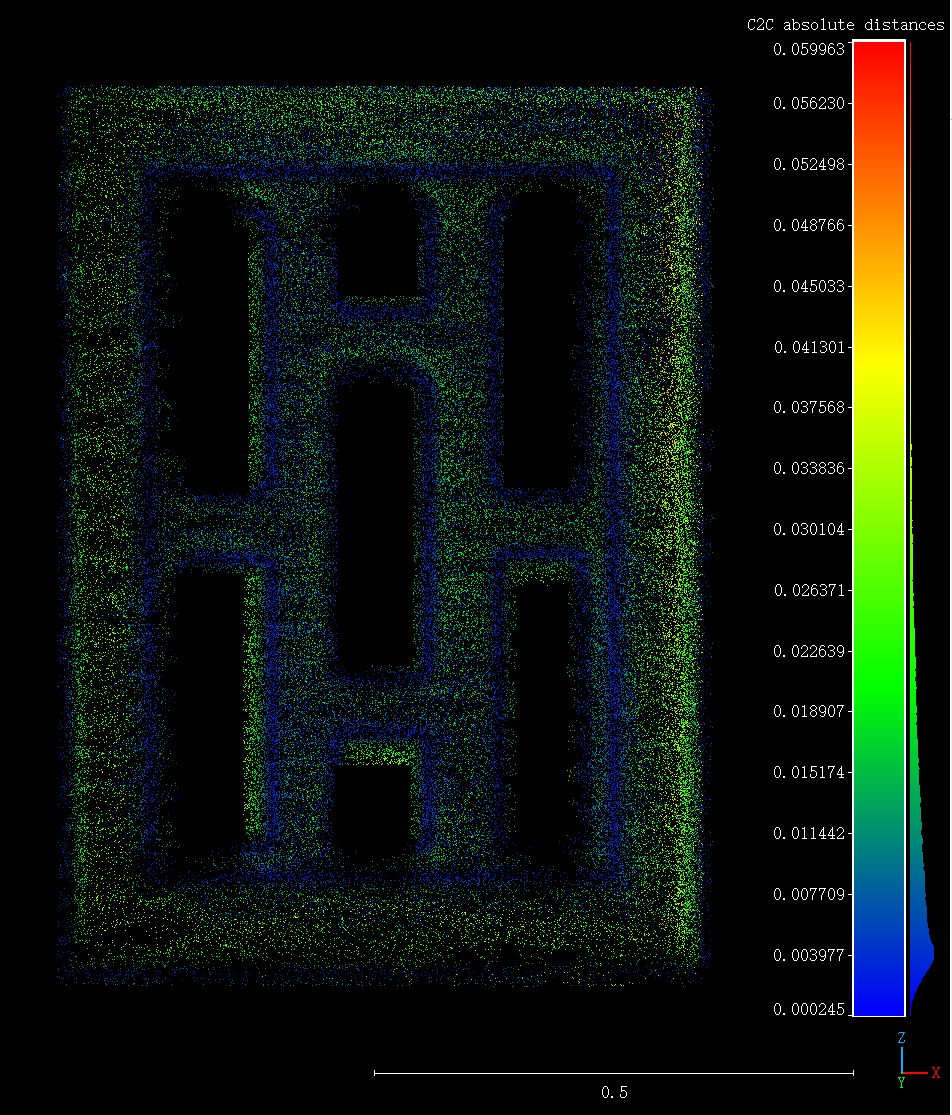


Fig. S4. Color-mapped deviation heatmap visualization.

## S2. Post-classification of reconstructed components

Description: This table summarizes reconstructed components by distinguishing point-cloud–supported elements from those inferred due to data loss, with engineering confidence levels (High/Moderate/Low) assigned based on geometric completeness and typological consistency.

| Component Type | Location | Evidence Used (Point Cloud / UAV / Drawing / Photo) | Reconstruction Status (Direct / Mixed / Inferred) | Confidence (High / Moderate / Low) | Notes |
| --- | --- | --- | --- | --- | --- |
| Roof ridge tiles (central segment) | Roof ridge | Sparse point cloud; UAV oblique images | Inferred | Low | Large-area loss at ridge; geometric form restored using typological reference of adjacent intact ridge tiles |
| Roof slope tiles (south-east slope) | Roof tiles | Dense, continuous point cloud | Direct | High | Tile rows preserved; point cloud clearly captures tile depth and alignment |
| Main beam (interior bay 2) | Interior | Dense point cloud with clear beam outline | Direct | High | Beam cross-section fully observable in LiDAR |
| Secondary timber purlin | Roof structure | Partial point cloud; adjacent structural continuity | Mixed | Moderate | Beam ends partially missing; length inferred based on parallel beams |
| Wall segment (north elevation) | Elevation | Continuous point cloud + CAD drawings | Direct | High | Masonry texture and thickness fully captured |
| Collapsed wall section (east side) | Damage table | Sparse point cloud; elevation drawing | Inferred | Low | Geometry reconstructed by aligning with typical masonry pattern in intact areas |
| Wooden window frame (east facade) | Window group | Partial point cloud + historical photos | Mixed | Moderate | Frame observed; decorative moulding inferred from photographed patterns |
| Stone window (south facade) | Window group | Complete point cloud + image | Direct | High | Stone frame fully present in LiDAR; details verified by images |
| Interior floor slab (bay 1) | Floor damage | Large missing area; structural module reference | Inferred | Low | No point cloud for slab surface; restored using typical bay spacing and structural grid |
| Timber beam–column joint | Interior | Partial point cloud + structural typology | Mixed | Moderate | Joint geometry obscured; proportions inferred from standard timber framing practice |

Table S2. Post-classification of reconstructed components.
